# Supplementary material for: Pro-oxidant response and accelerated ferroptosis caused by synergetic Au(I) release in hypercarbon-centered gold(I) cluster prodrugs
Source: Nat Commun. 2022 Aug 9;13:4669. doi: 10.1038/s41467-022-32474-y (PMC9363434; doi:10.1038/s41467-022-32474-y)
Supplement: Supplementary file 1 — Supplementary Information [file 41467_2022_32474_MOESM1_ESM.pdf]

Supplementary Information

Pro-oxidant Response and Accelerated Ferroptosis Caused by Synergetic Au(I) Release in Hypercarbon-centered Gold(I) Cluster Prodrugs

Kui Xiao,[1] Niyuan Zhang,[2] Feifei Li,[3] Dayong Hou,[2,4,5] Xiaoyi Zhai,[1] Wanhai Xu,[4,5*] Gelin Wang,[3*] Hao Wang,[2*] Liang Zhao[1*]

[1] Key Laboratory of Bioorganic Phosphorus Chemistry and Chemical Biology (Ministry of Education), Department of Chemistry, Tsinghua University, Beijing 100084, China.

[2] CAS Key Laboratory for Biomedical Effects of Nanomaterials and Nanosafety, CAS Center for Excellence in Nanoscience, National Center for Nanoscience and Technology (NCNST), Beijing 100190, China.

[3] School of Pharmaceutical Sciences, Tsinghua-Peking Joint Center for Life Sciences, Tsinghua University, Beijing, 100084, China

[4] Department of Urology, the Fourth Hospital of Harbin Medical University, Heilongjiang Key Laboratory of Scientific Research in Urology, Harbin 150001, China.

[5] NHC Key Laboratory of Molecular Probes and Targeted Diagnosis and Therapy, Harbin Medical University, Harbin 150001, China.

Supplementary Figures

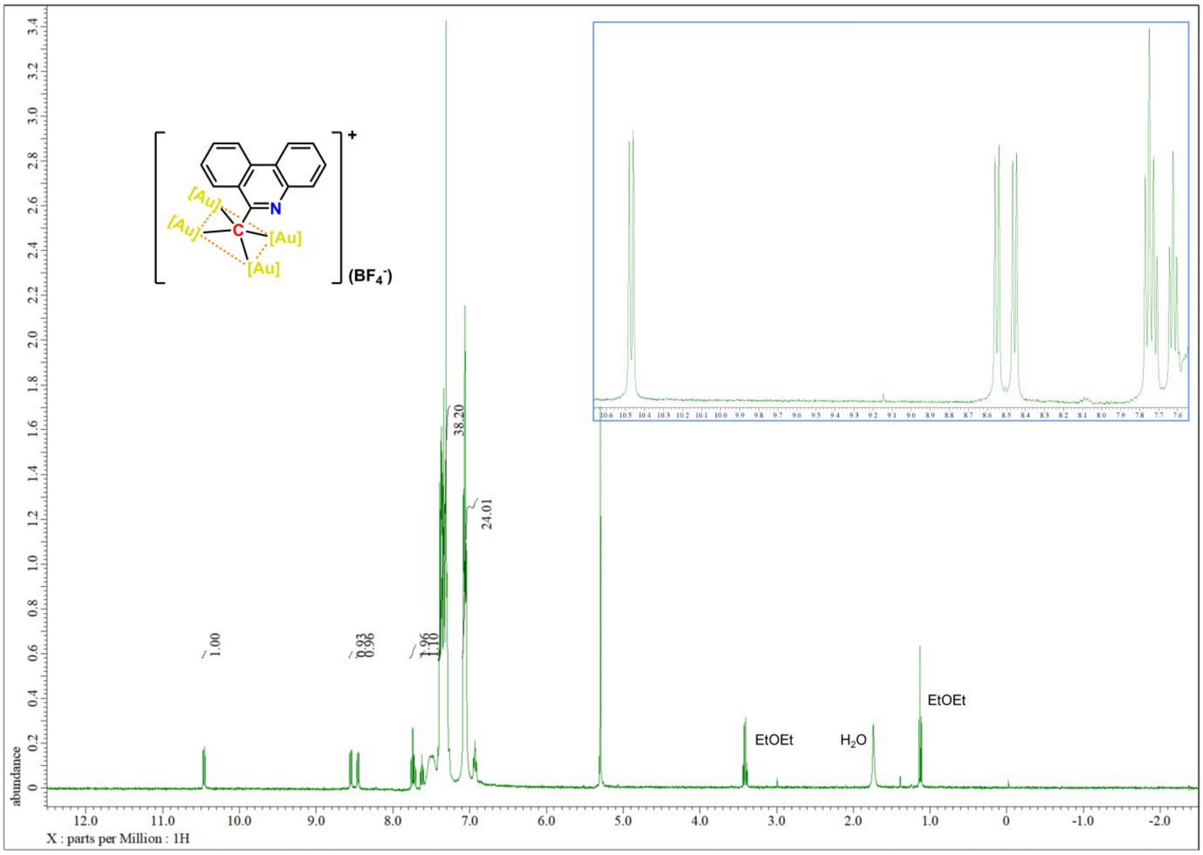

Supplementary Figure S1 ^{1}H NMR spectrum of **PAA4** (400 MHz, DMSO-d$_6$, 298K).

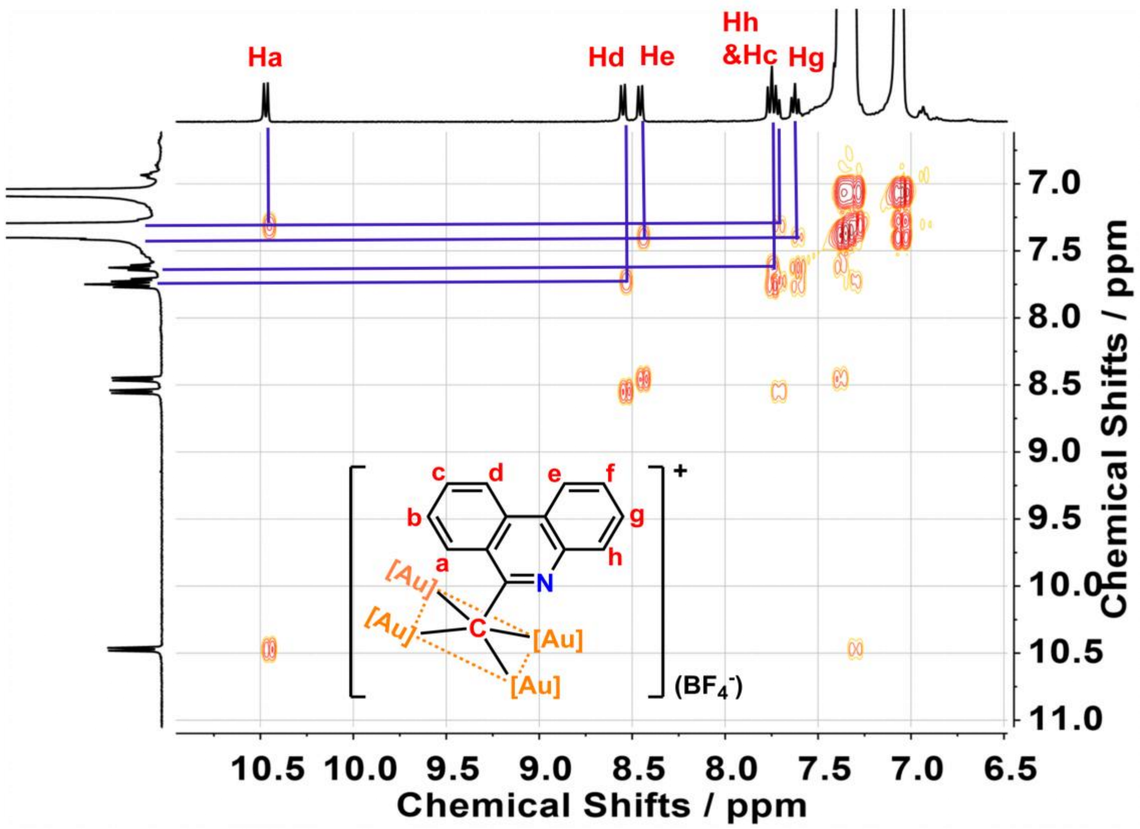

Supplementary Figure S2 H-H COSY spectrum of **PAA4** (CD_2Cl_2, 298 K).

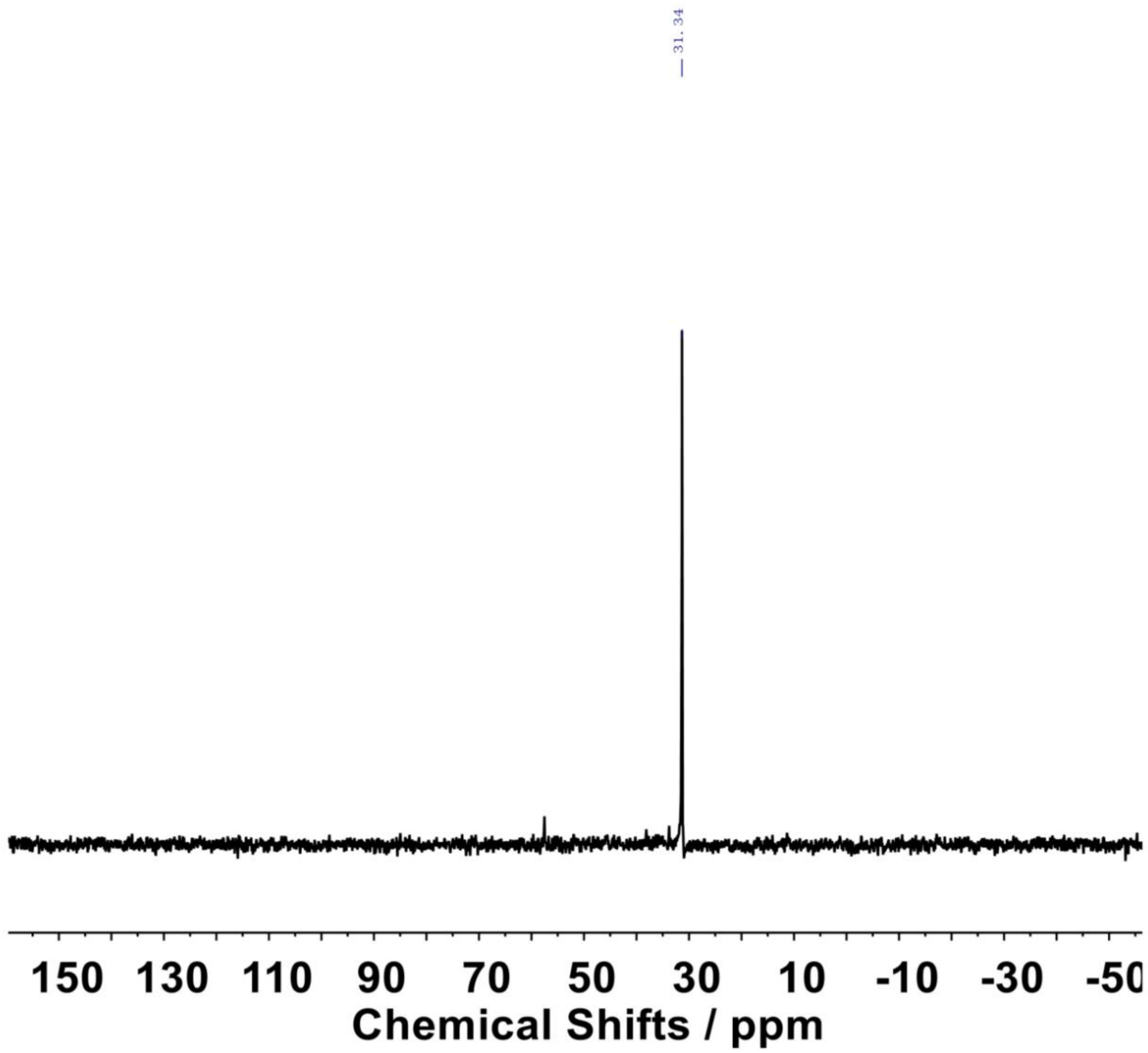

Supplementary Figure S3 ^{31}P NMR spectrum of **PAA4** (162 MHz, CD_2Cl_2, 298K).

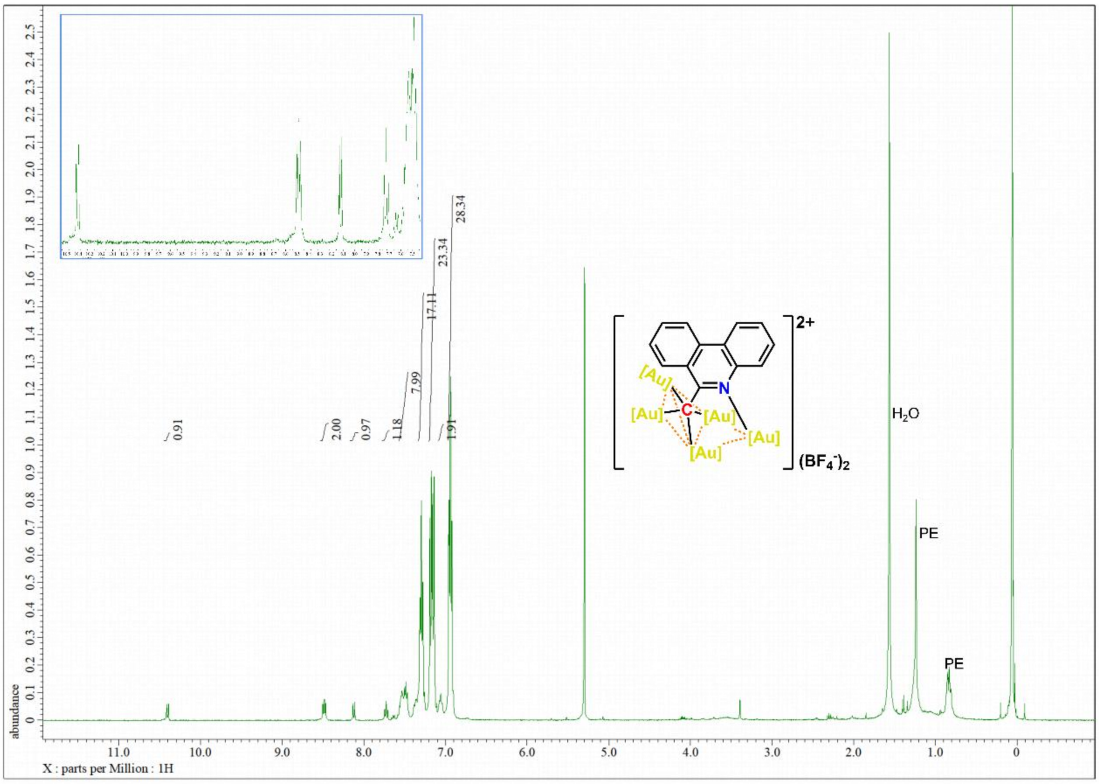

Supplementary Figure S4 ^{1}H NMR spectrum of **PAA5** (400 MHz, CD_2Cl_2, 298K).

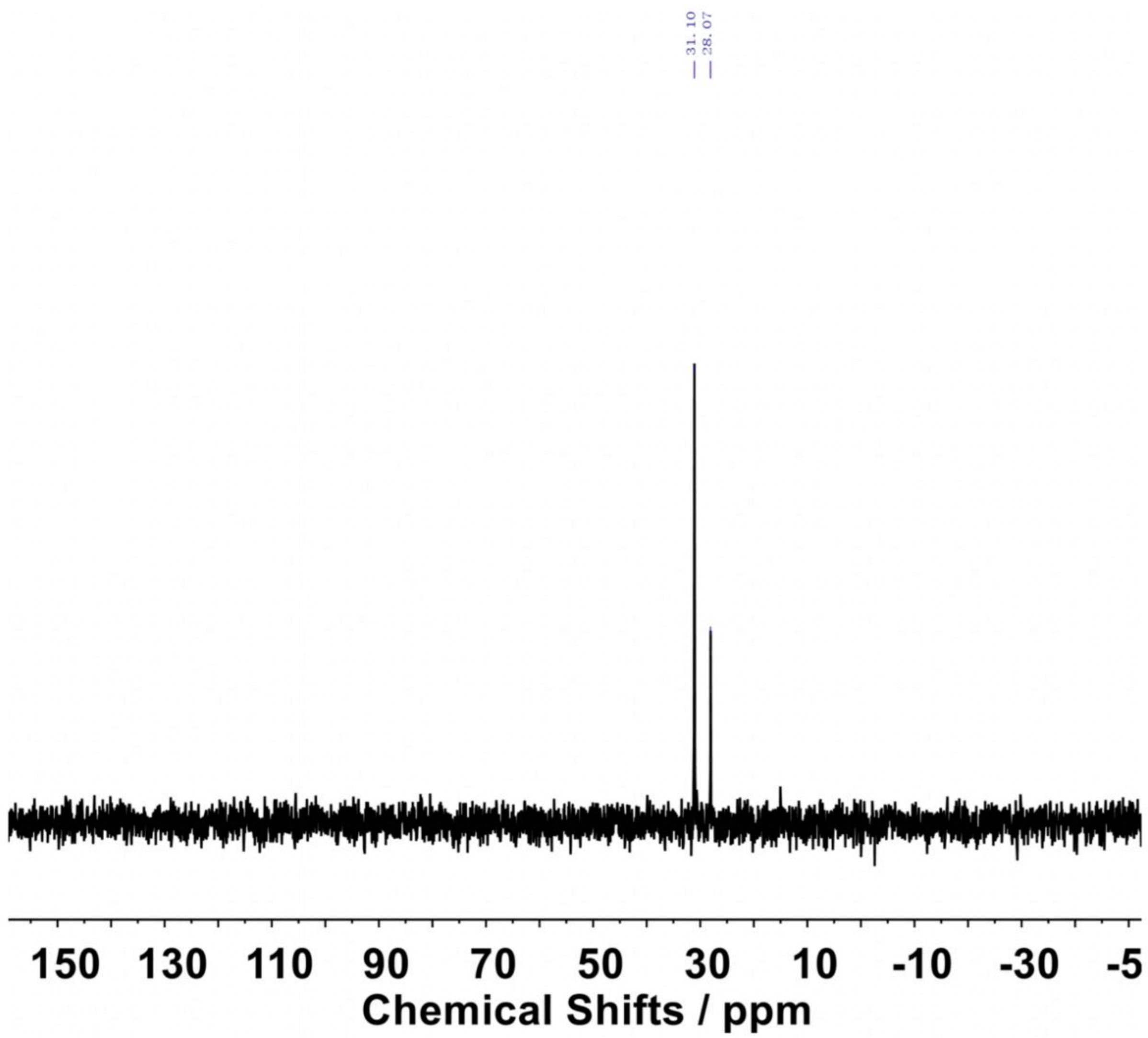

Supplementary Figure S5 ^{31}P NMR spectrum of **PAA5** (162 MHz, CD_2Cl_2, 298K).

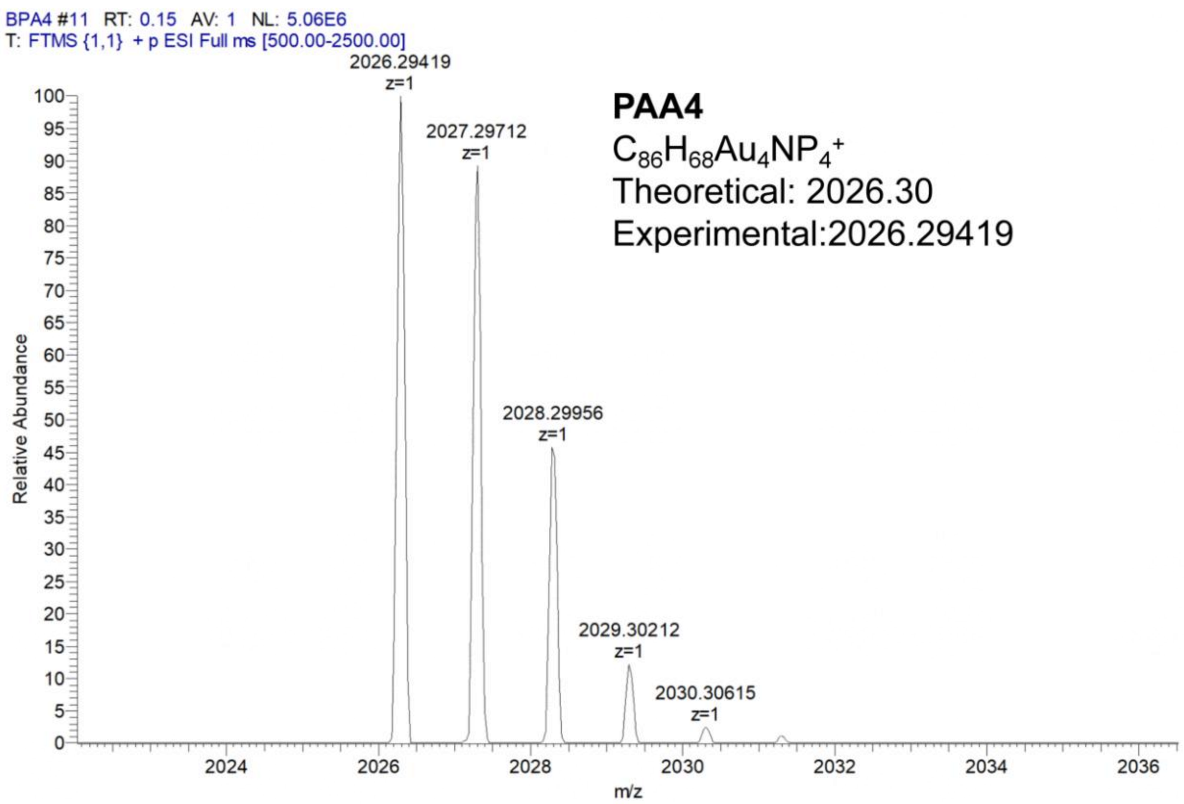

Supplementary Figure S6 ESI-MS spectra of **PAA4**.

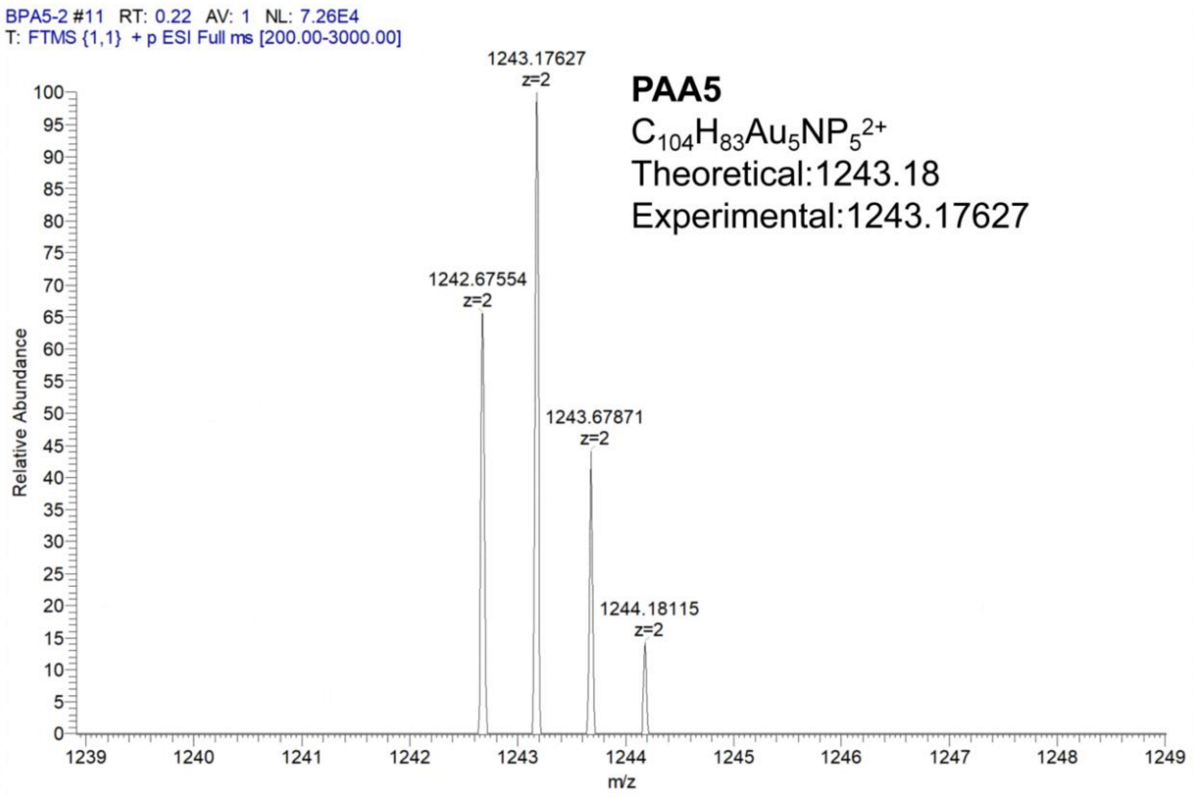

Supplementary Figure S7 ESI-MS spectrum of **PAA5**.

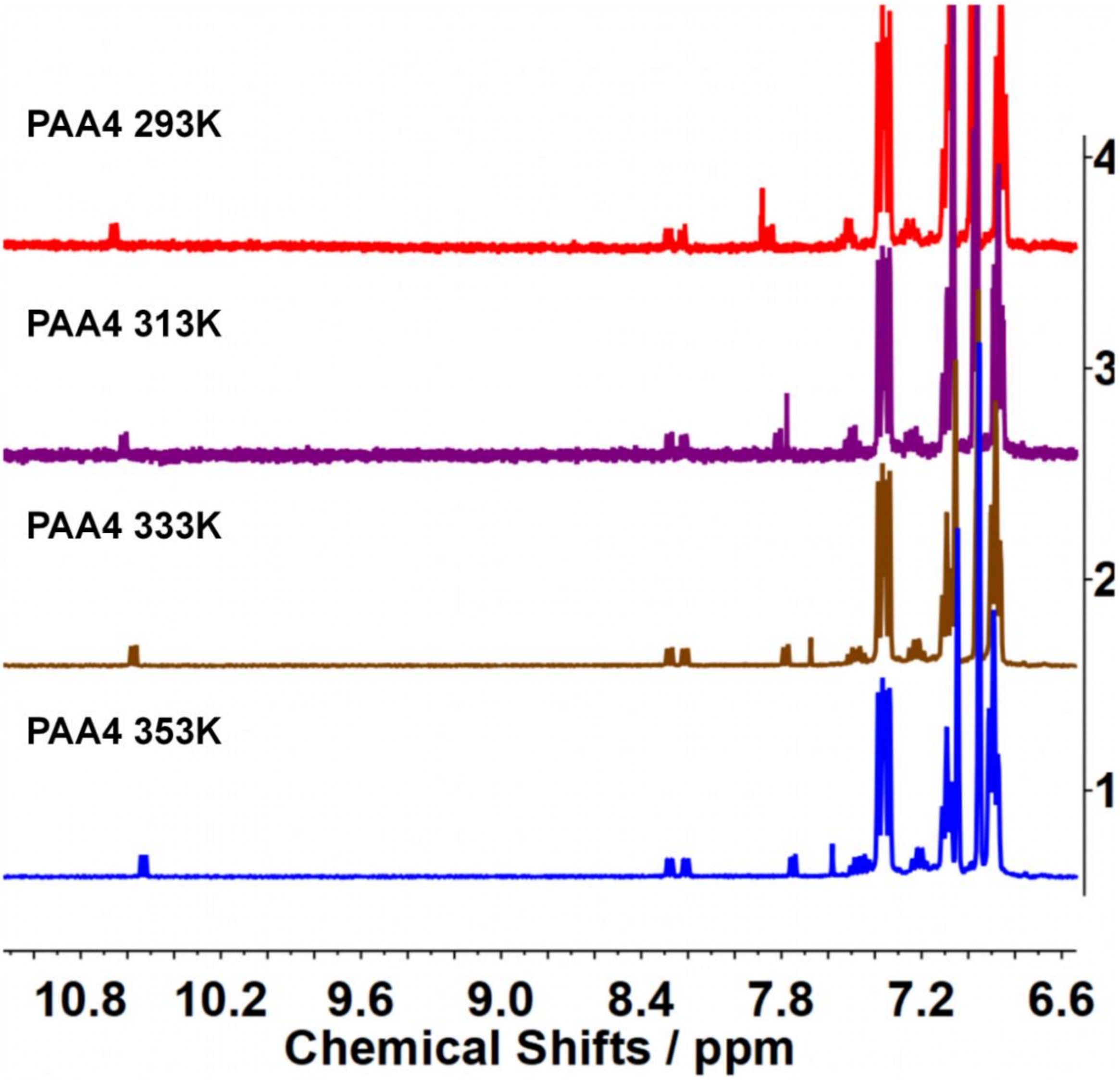

Supplementary Figure S8 ^1H-NMR spectra (toluene-d_8 : DMSO-d_6 = 9:1, 298 K) of **PAA4** from 293 to 353 K, suggesting the good thermal stability of **PAA4**.

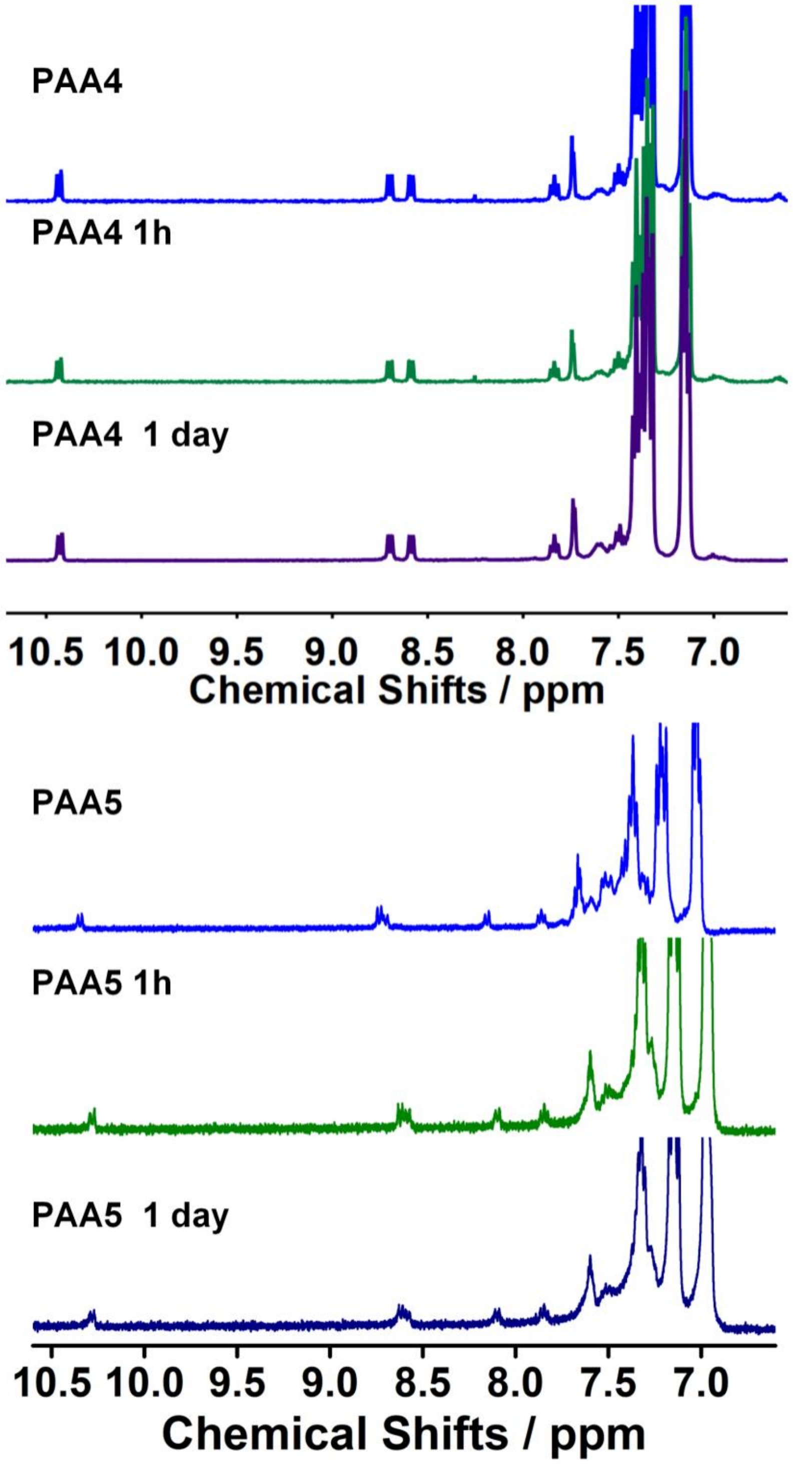

Supplementary Figure S9 ^{1}H-NMR spectra (DMSO-d$_6$: D_2O = 9:1, 298 K) of **PAA4** (top) and **PAA5** (down) in one day, confirming their good stability upon exposure to moisture.

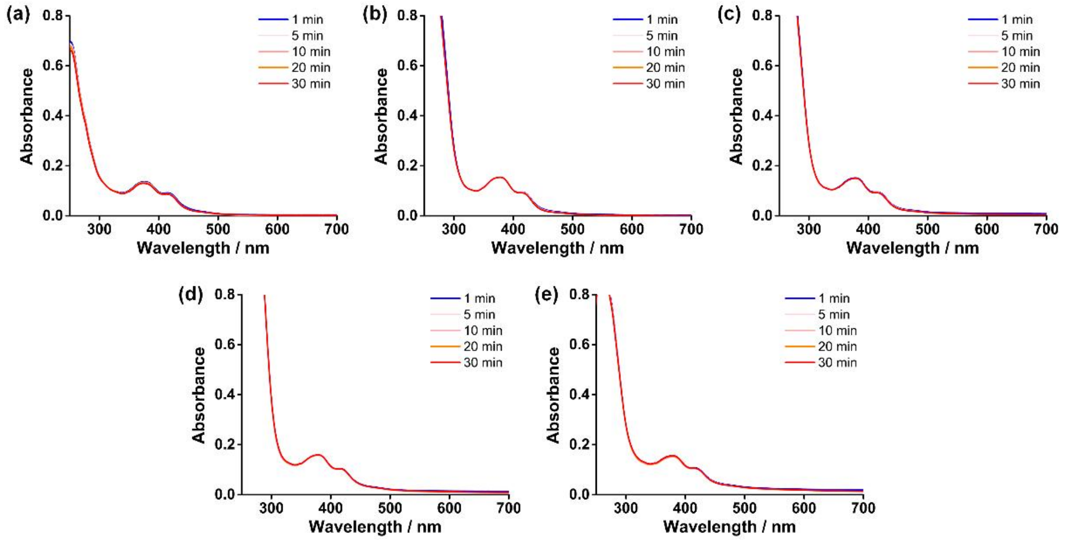

Supplementary Figure S10 UV-vis spectral monitoring on (a-e) **PAA4** (1.0×10^{-5} M, DMSO : H_2O = 1:1, 298 K) in the PBS buffer ([Na_2HPO_4] + [KH_2PO_4] = 0.01 M), (a) pH = 4.9, (b) pH = 5.9, (c) pH = 6.8, (d) pH = 7.7, (e) pH = 8.7, suggesting the good stability of **PAA4** in a wide pH range. (n = 2 independent experiments).

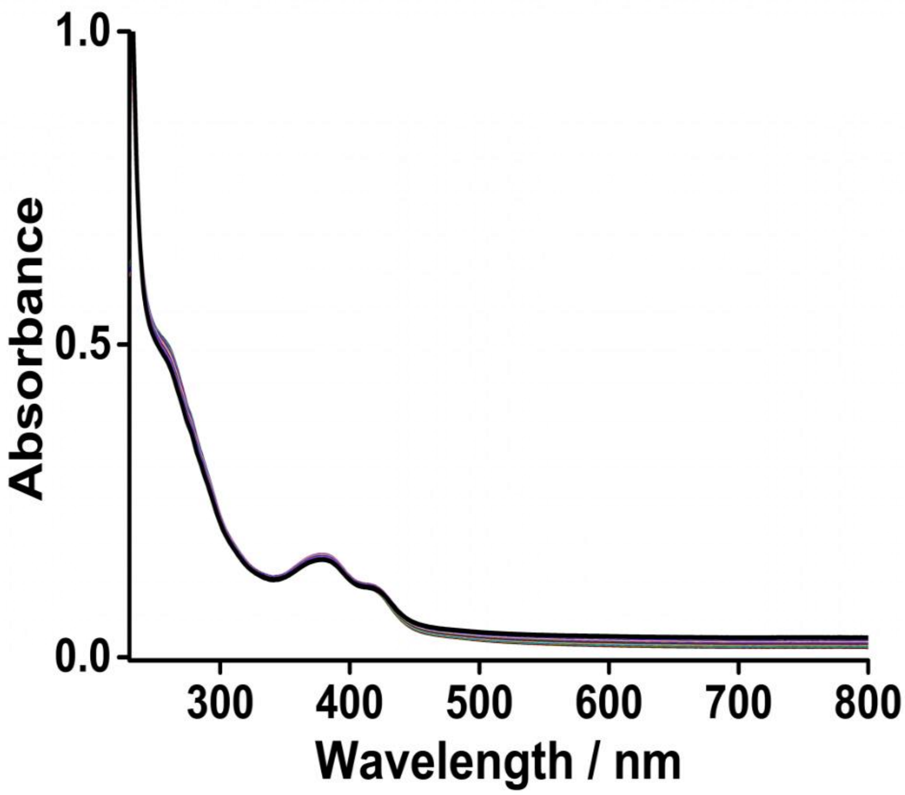

Supplementary Figure S11 The good stability of **PAA4** in dulbecco's modified eagle medium (DMEM) (**PAA4** = 1.0 × 10^{-5} M, 298 K, 1.0h). (n = 2 independent experiments).

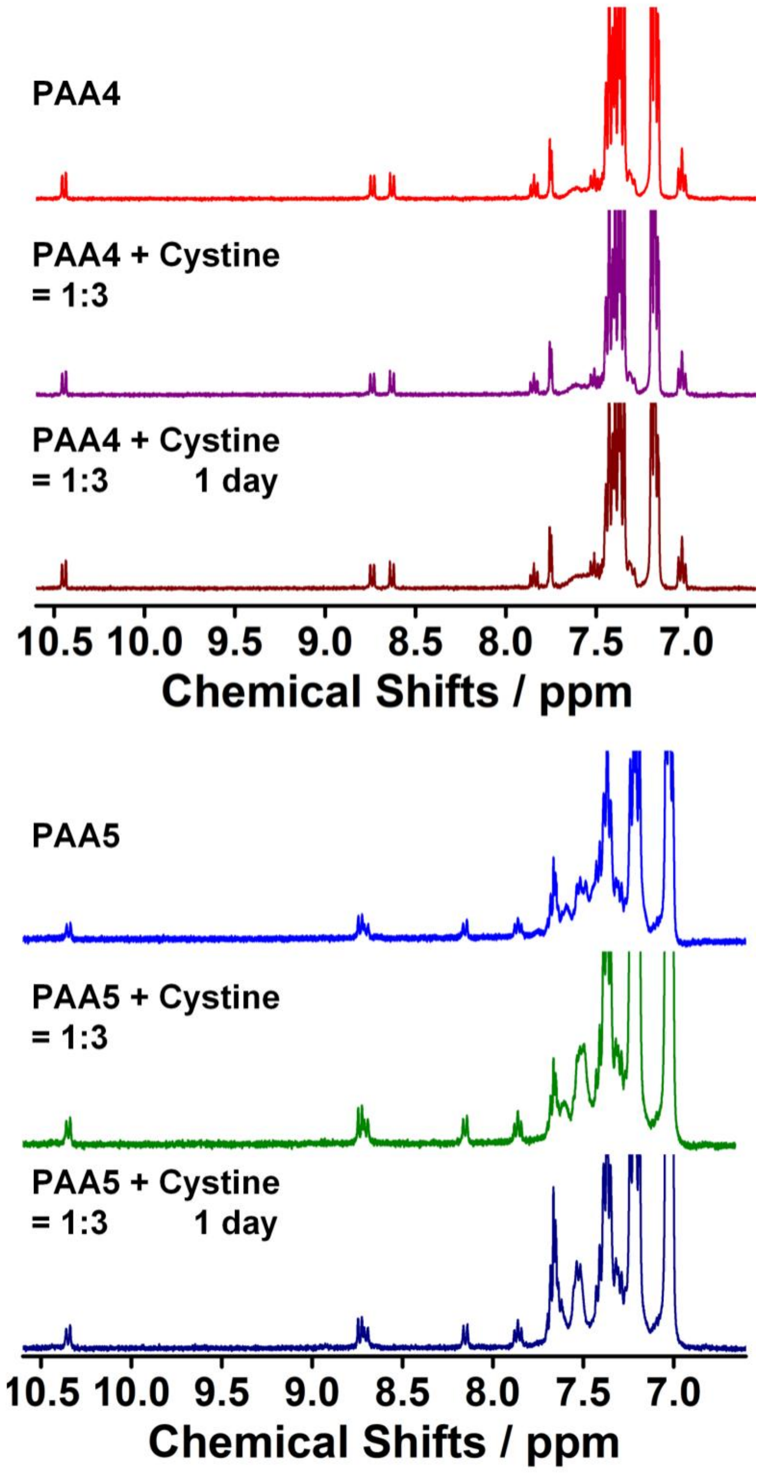

Supplementary Figure S12 The good stability of **PAA4** and **PAA5** towards cystine. Top: ^{1}H-NMR spectra monitoring of **PAA4** : cystine = 1 : 3 in one day (DMSO-d_6 : D_2O = 9:1, 298 K). Down: ^{1}H-NMR spectra of **PAA5** : cystine = 1 : 3 in one day (DMSO-d_6 : D_2O = 9:1, 298 K).

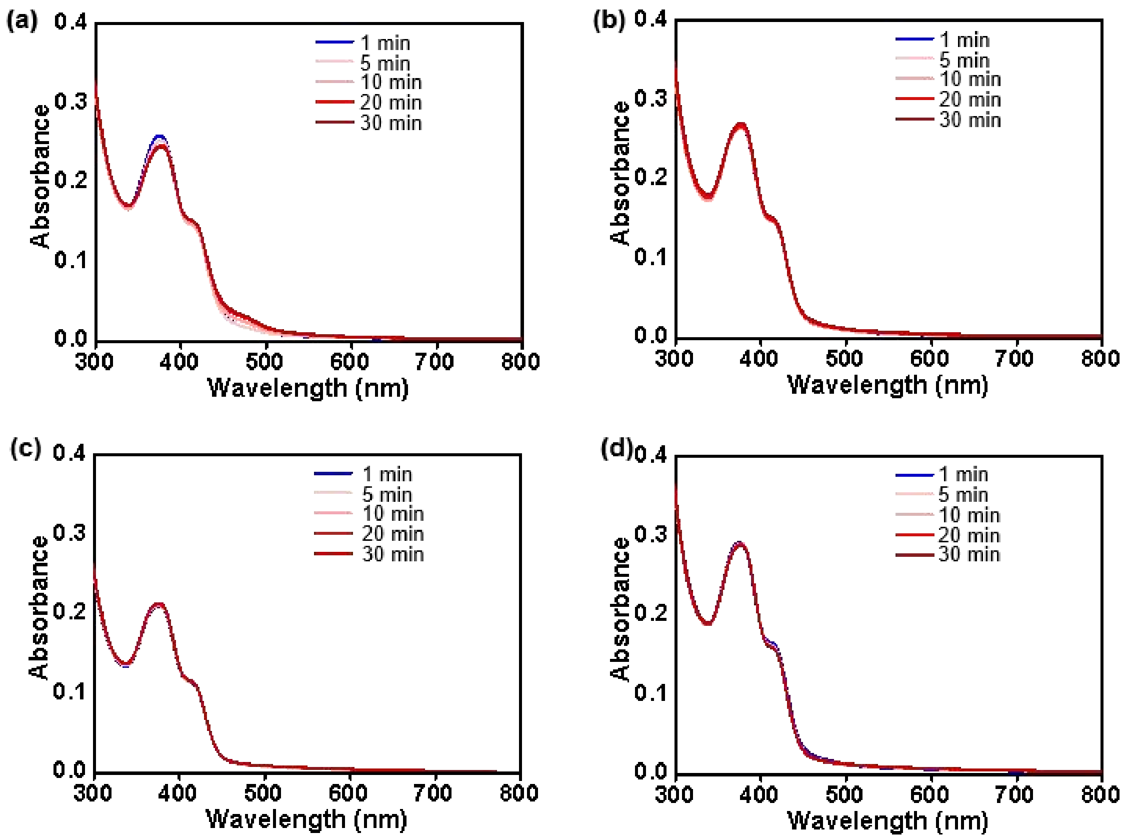

Supplementary Figure S13 UV-vis spectral monitoring on (a-d) **PAA4** (1.0×10^{-5} M, DMSO : H_2O = 1:1, 298 K) in the PBS buffer ([Na_2HPO_4] + [KH_2PO_4] = 0.01 M), (a) pH = 5.9, (b) pH = 6.8, (c) pH = 7.7, (d) pH = 8.7, suggesting the good stability of **PAA5** in a wide pH range. (n = 2 independent experiments).

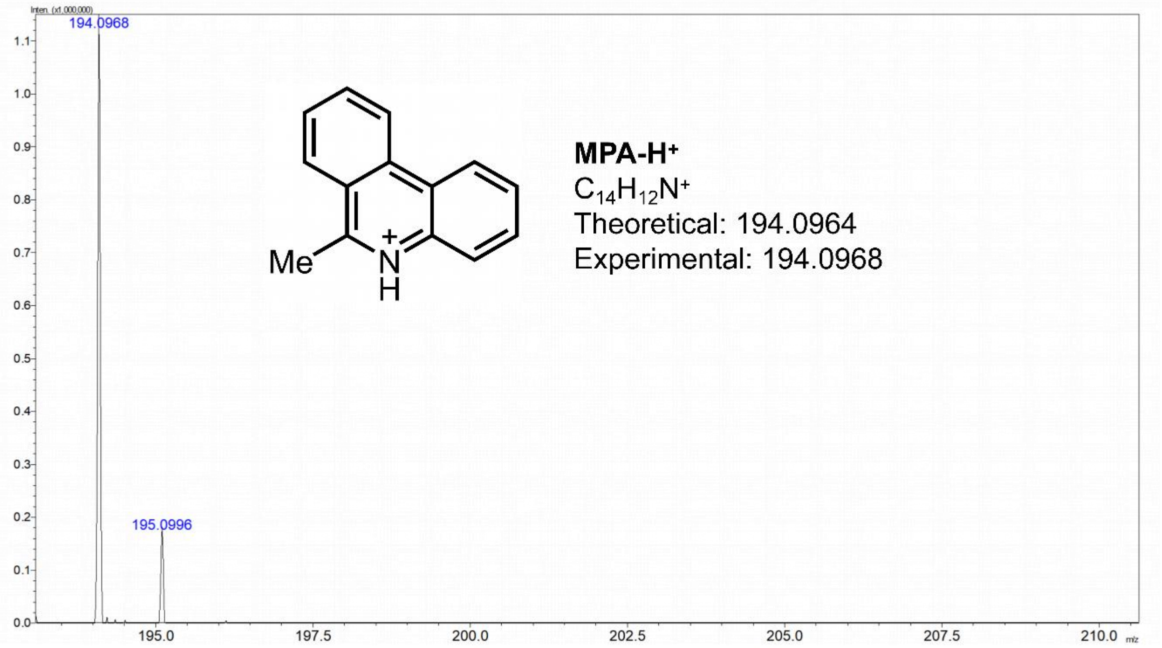

Supplementary Figure S14 ESI-MS of MPA.

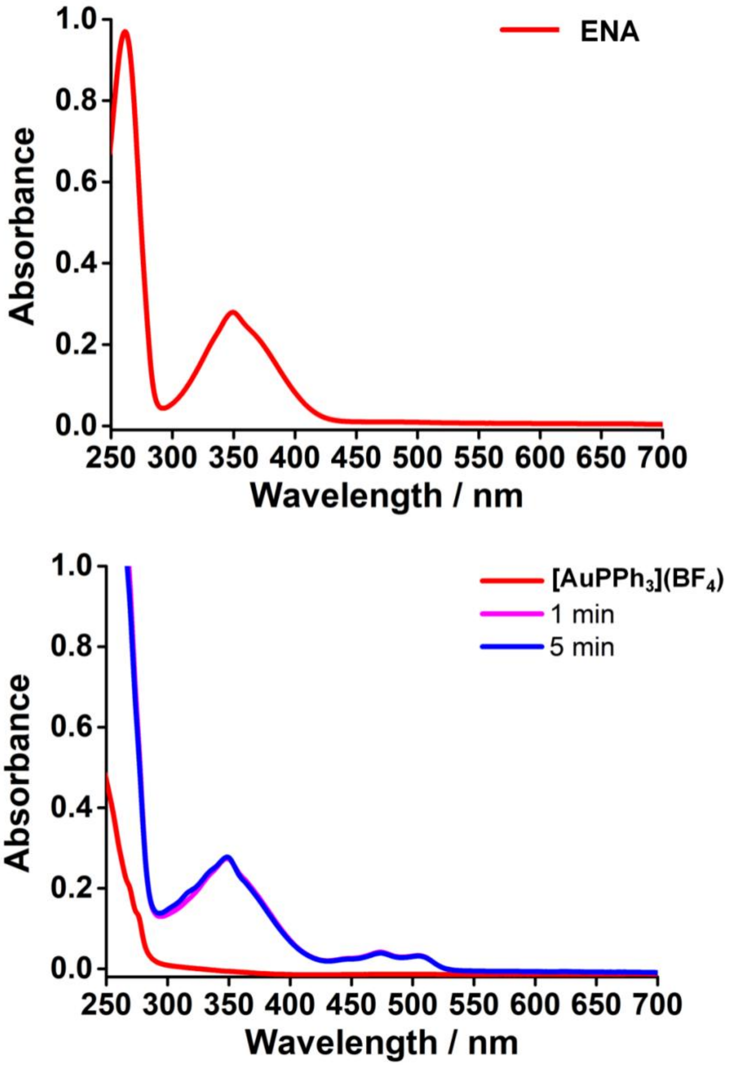

Supplementary Figure S15 Reaction of **ENA** with $[AuPPh_3]BF_4$ to generate **MBIA** monitored by UV-vis spectra. (**A**) UV-vis spectra of **ENA** (4.0×10^{-5} M, DMSO : H_2O = 1:1, 298 K). (**B**) UV-vis spectra monitoring of the reaction mixture of **ENA** : $[AuPPh_3]BF_4$ = 1 : 1 (**ENA** = 4.0×10^{-5} M, DMSO : H_2O = 1:1, 298 K) within 5 min. (n = 2 independent experiments).

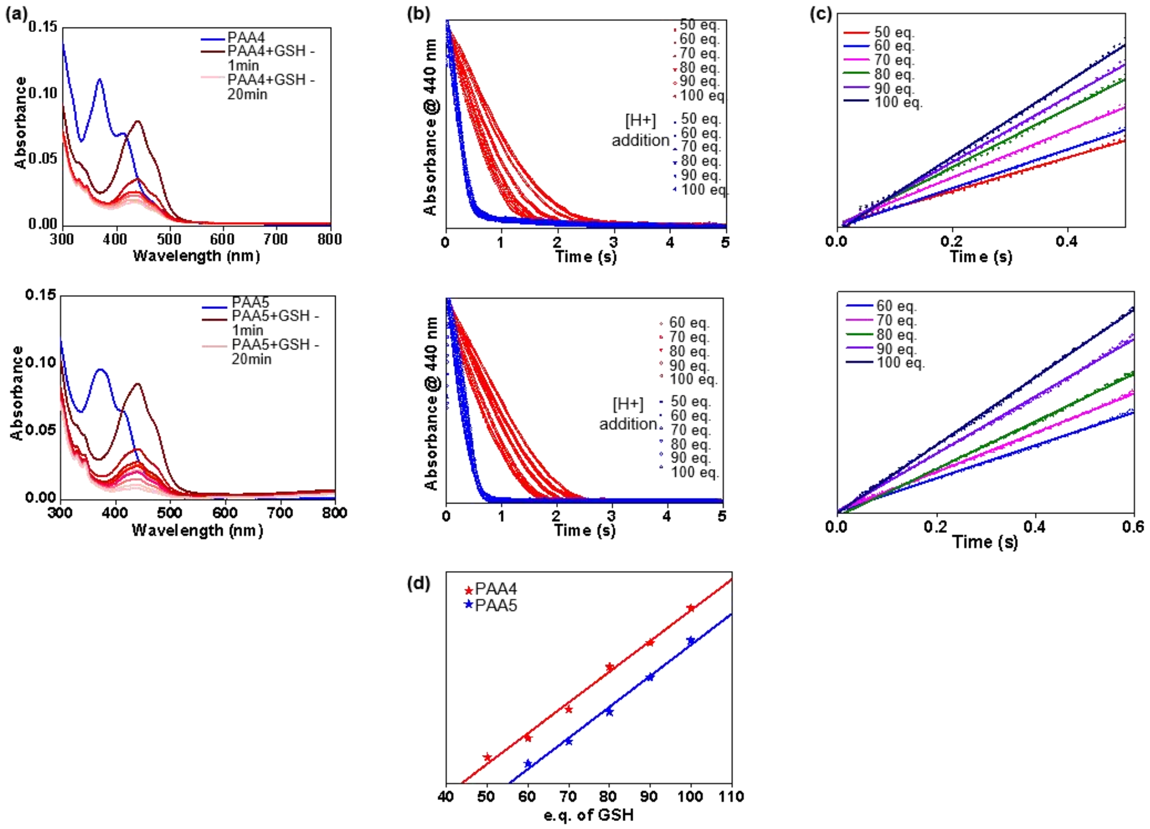

Supplementary Figure S16 (**a**) UV-vis spectral monitoring on the reaction mixture of (up) **PAA4**:GSH = 1:10, (down) **PAA5**:GSH = 1:10 ($c_{PAA4} = c_{PAA5} = 5.0 \times 10^{-4}$ M, MeOH, 298 K). (**b**) UV-vis spectral monitoring at 440 nm on the reaction mixture of (up) **PAA4**:GSH = 1:50 to 1:100, (down) **PAA5**:GSH = 1:50 to 1:100 ($c_{PAA4} = c_{PAA5} = 5.0 \times 10^{-4}$ M, MeOH, 298 K), with red line GSH:[HBF_4] = 1:0 or blue line GSH:[HBF_4] = 1:1 (pH = 3.5). (**c**) Lines fitting of the absorbance decline curves of (up) **PAA4**:GSH = 1:50 to 1:100, (down) **PAA5**:GSH = 1:60 to 1:100, ($c_{PAA5} = 5.0 \times 10^{-4}$ M, MeOH, 298 K). (**d**) Fitting with the Arrhenius equation. (n = 3 independent experiments).

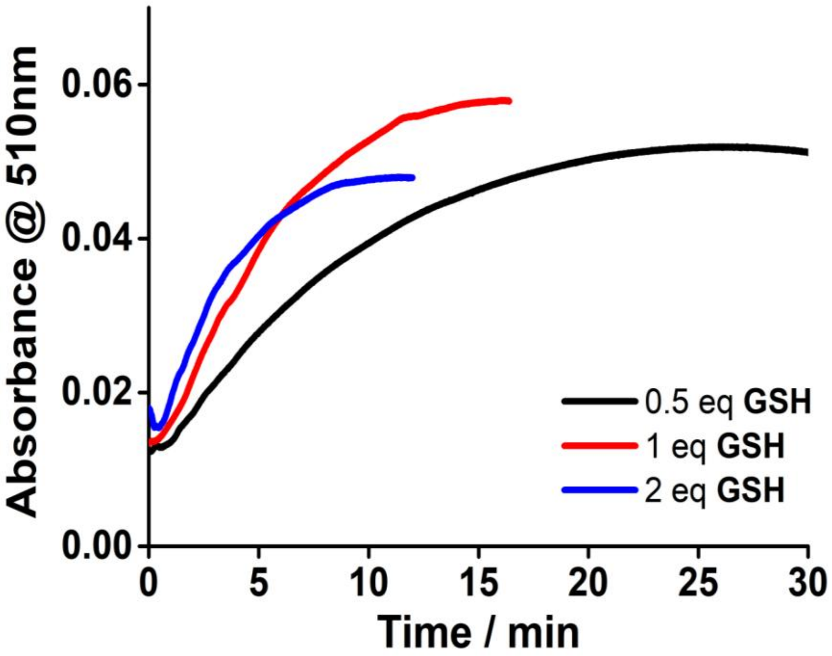

Supplementary Figure S17 UV-vis spectral monitoring on **MBIA** generated in the reaction mixture of **PAA4**, **ENA** and **GSH**. Absorbance variation curves at 510 nm corresponding to the reaction mixtures of **PAA4** : GSH : **ENA =** 1 : 0.5 : 4, 1 : 1 : 4 and 1 : 2 : 4 (**PAA4** = 1.0 × 10^{-5} M, DMSO : H_2O = 1:1, 298 K). (n = 2 independent experiments).

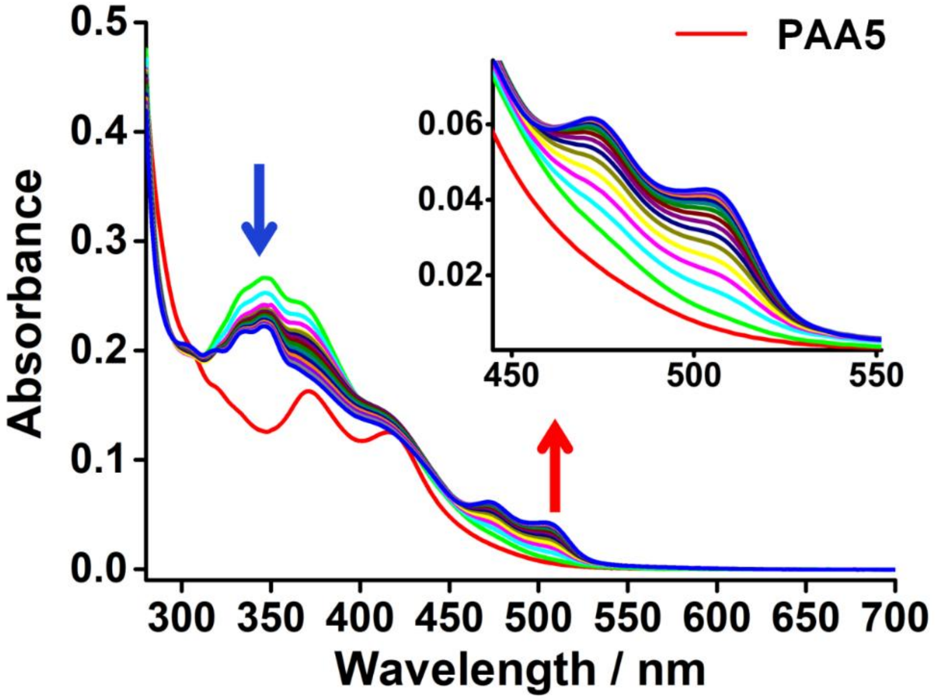

Supplementary Figure S18 UV-vis spectra monitoring of the reaction mixtures of **PAA5**, **ENA** and **GSH**. UV-vis spectra monitoring on the reaction mixture of **PAA5** : **ENA** : GSH = 1 : 4 : 0.5 (**PAA5** = 1.0×10^{-5} M, DMSO : H_2O = 1:1, 298 K) within 20 min. Insert: UV-vis spectra monitoring of **MBIA**. (n = 2 independent experiments).

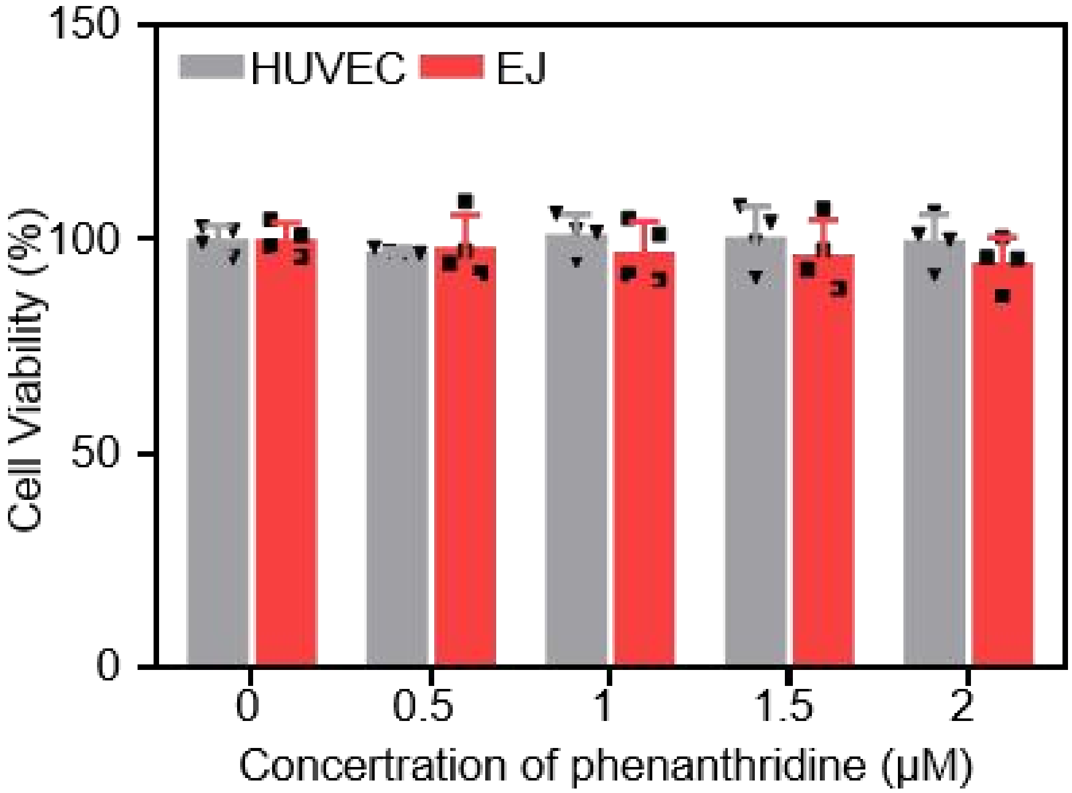

Supplementary Figure S19 *In vitro* antitumor activity evaluation of phenanthridine. Cell viability assay of HUVEC and EJ cells treated with a series of concentrations of phenanthridine for 24 h. Phenanthridine reveals no significant cytotoxicity in EJ and HUVEC cell lines.Data were expressed as mean ± SD (n = 3 repeat wells). Source data are provided as a Source Data file.

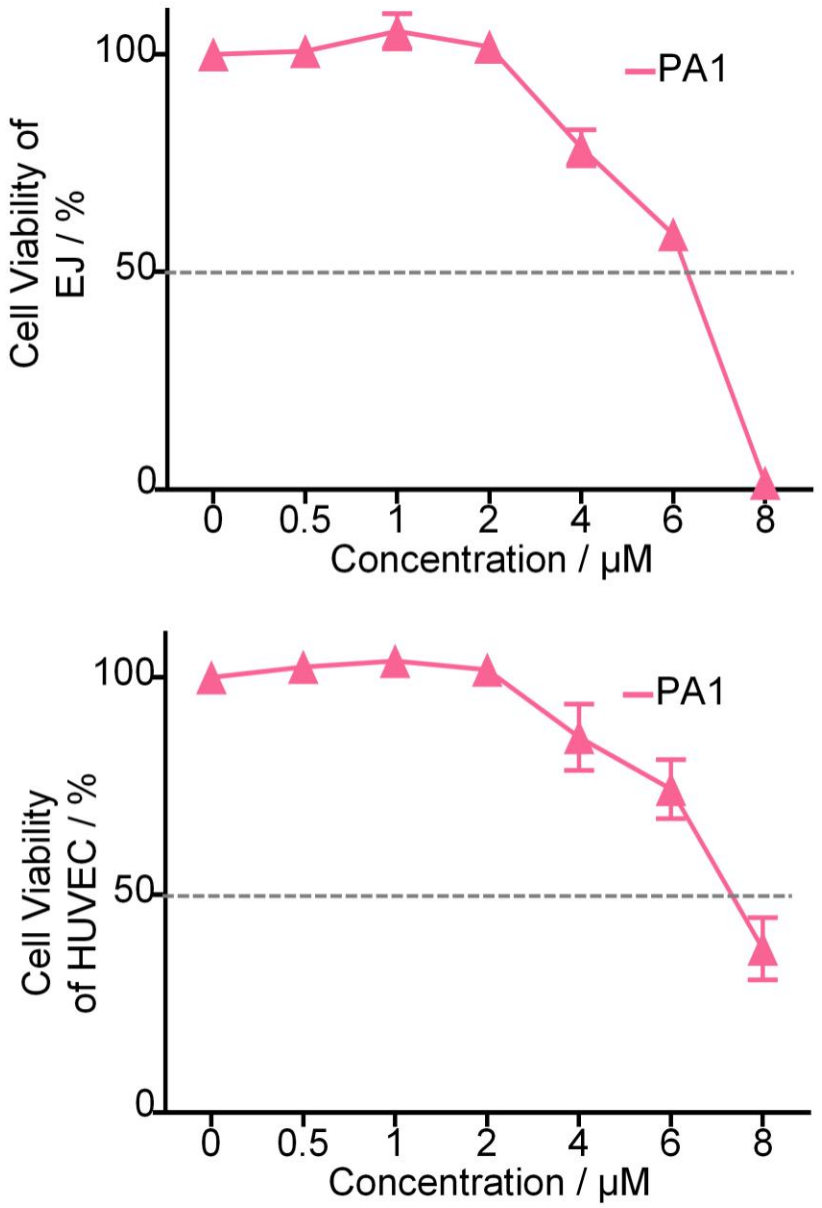

Supplementary Figure S20 *In vitro* antitumor activity evaluation of **PA1** (IC50 6.0 μM for EJ cells and 7.3 μM for HUVEC cells). Cell viability assay of HUVEC and EJ cells treated with a series of concentrations of **PA1** for 24 h. Data were expressed as mean ± SD (n = 3 independent experiments examined over triplicates). Source data are provided as a Source Data file.

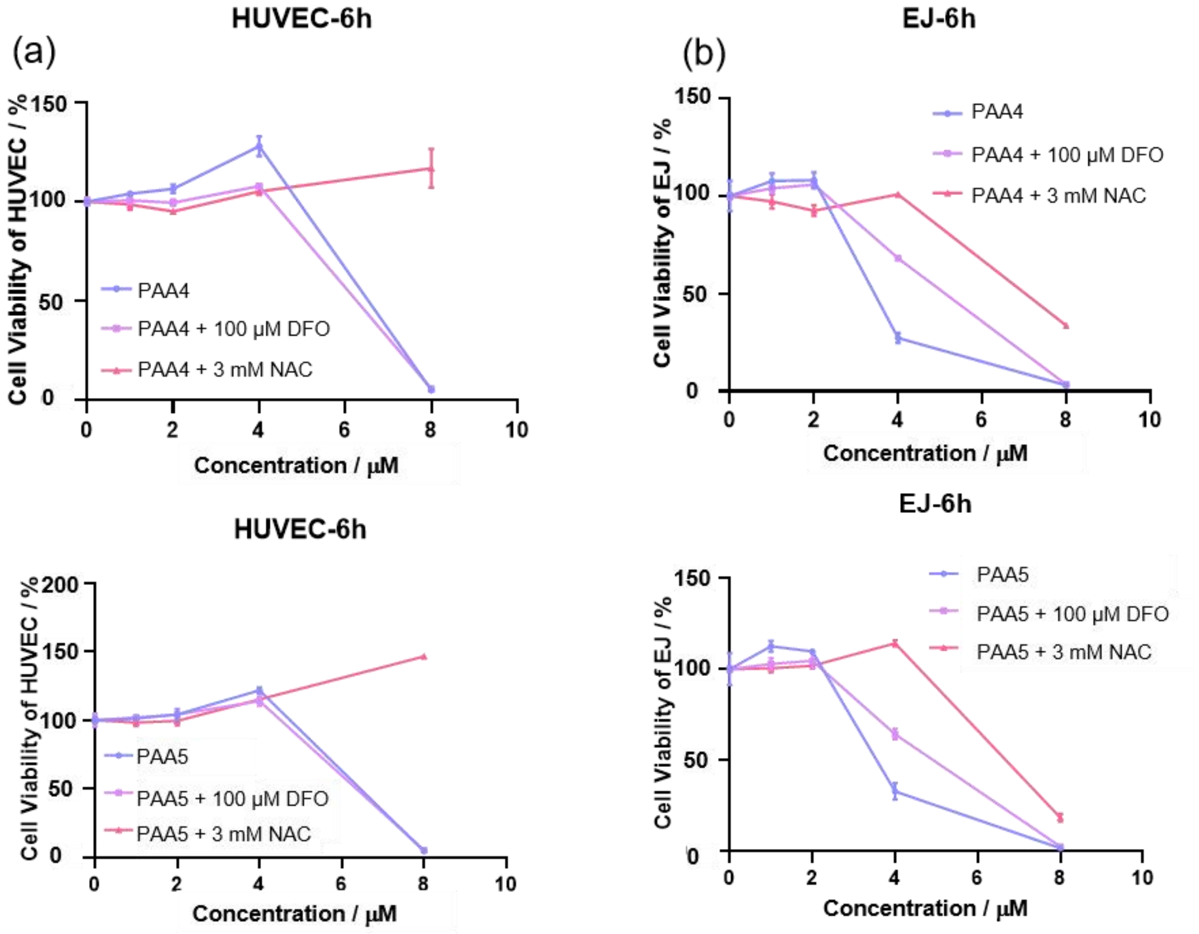

Supplementary Figure S21 Cell viability assay of HUVEC (a) and EJ (b) cells treated with **PAA4** (up) and **PAA5** (b) for 6 h with or without 100μM DFO or 3.0 mM NAC pretreatment for 2 hours. Data were expressed as mean ± SD (n = 3 independent experiments examined over triplicates). Source data are provided as a Source Data file.

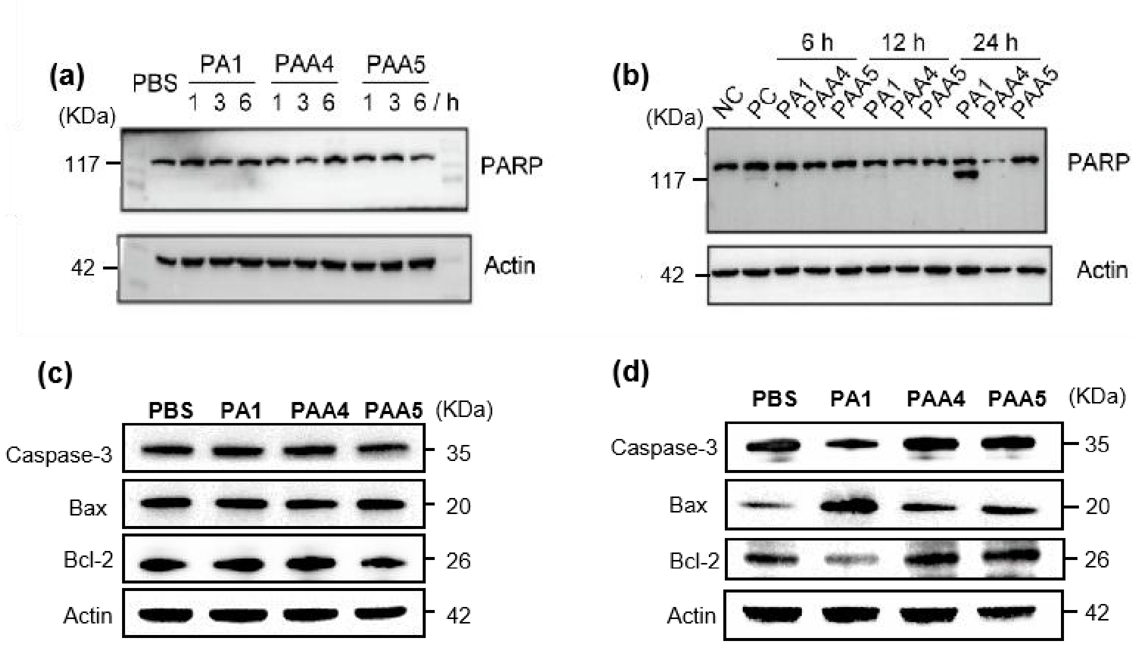

Supplementary Figure S22 Western blot analysis of PARP cleavage in EJ cells (a) and HUVEC cells (b) treated by 8.0 μM **PA1**, 4.0 μM **PAA4** or 4.0 μM **PAA5**, respectively. Western blot analysis of caspase 3 activation and Bax pathway in EJ cells (c) treated by 6h 8.0 μM **PA1**, 4.0 μM **PAA4** or 4.0 μM **PAA5**, and HUVEC cells (d) treated by 24h 8.0 μM **PA1**, 4.0 μM **PAA4** or 4.0 μM **PAA5**, respectively.Apoptosis is only observed in **PA1**-treated HUVEC cells after 24h. n = 2 independent experiments. Source data are provided as a Source Data file.

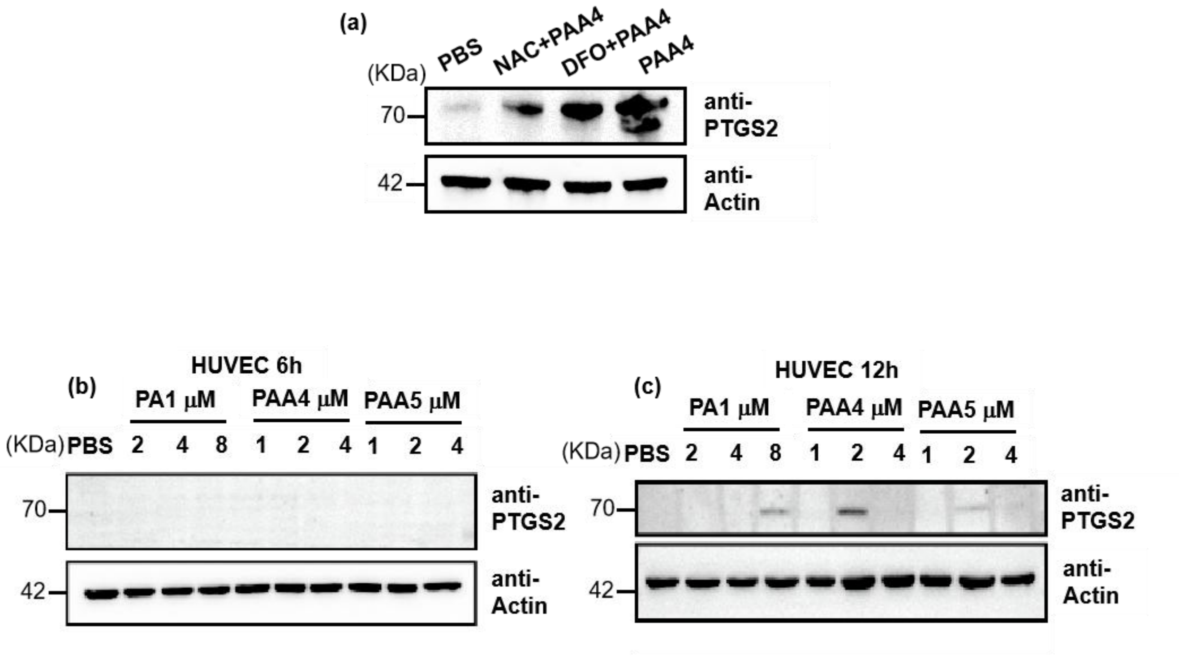

Supplementary Figure S23 (a) Western blot analysis of PTGS2 protein expression in EJ cells treated by 4.0 μM **PAA4** for 4h. (b) and (c) Western blot analysis of PTGS2 protein expression in HUVEC cells treated by **PA1**, **PAA4** or **PAA5** for 6h or 12h, respectively. PTGS2 protein expression was found in **PAA4**-treated EJ cells. n = 2 independent experiments. Source data are provided as a Source Data file.

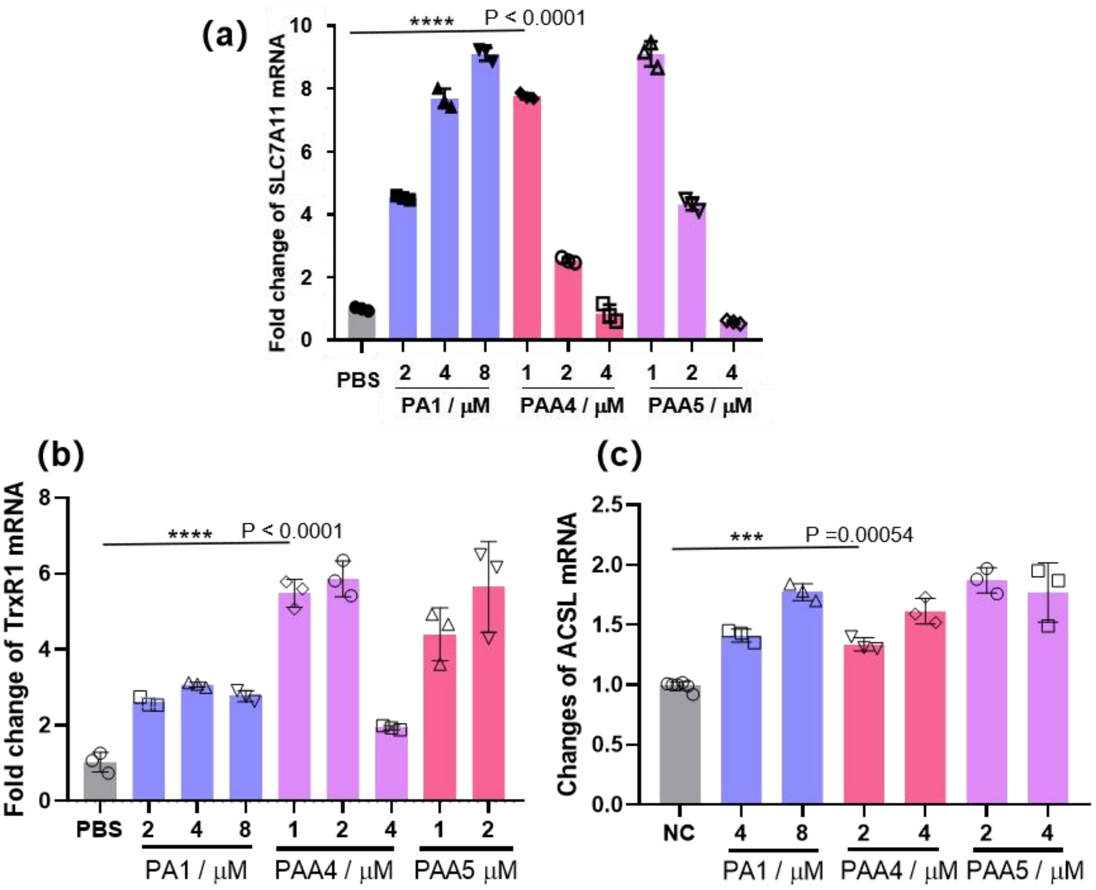

Supplementary Figure S24 Changes of (a) SLC7A11 mRNA, (b) TrxR1 mRNA and (c) ACSL mRNA, in EJ cells treated by **PAA4**, **PAA5** or **PA1** for 6 hours. Asterisks (*) denote the statistical significance: 0.01< * $P < 0.05$, 0.001< ** $P \leq 0.01$, 0.0001< *** $P \leq 0.001$, **** $P \leq 0.0001$, P values were performed with one-way ANOVA followed by post hoc Tukey's test. Data were expressed as mean ± SD (n = 3 independent experiments examined over triplicates). The SLC7A11 mRNA and TrxR1 mRNA levels in EJ cells treated by low dose of **PAA4** and **PAA5** increase due to enhanced oxidant stress within cells, while the reduction along with treatment by high dose of **PAA4** and **PAA5** is due to generation of ferroptosis. ACSL mRNA is slightly increased due to ferroptosis. Source data are provided as a Source Data file.

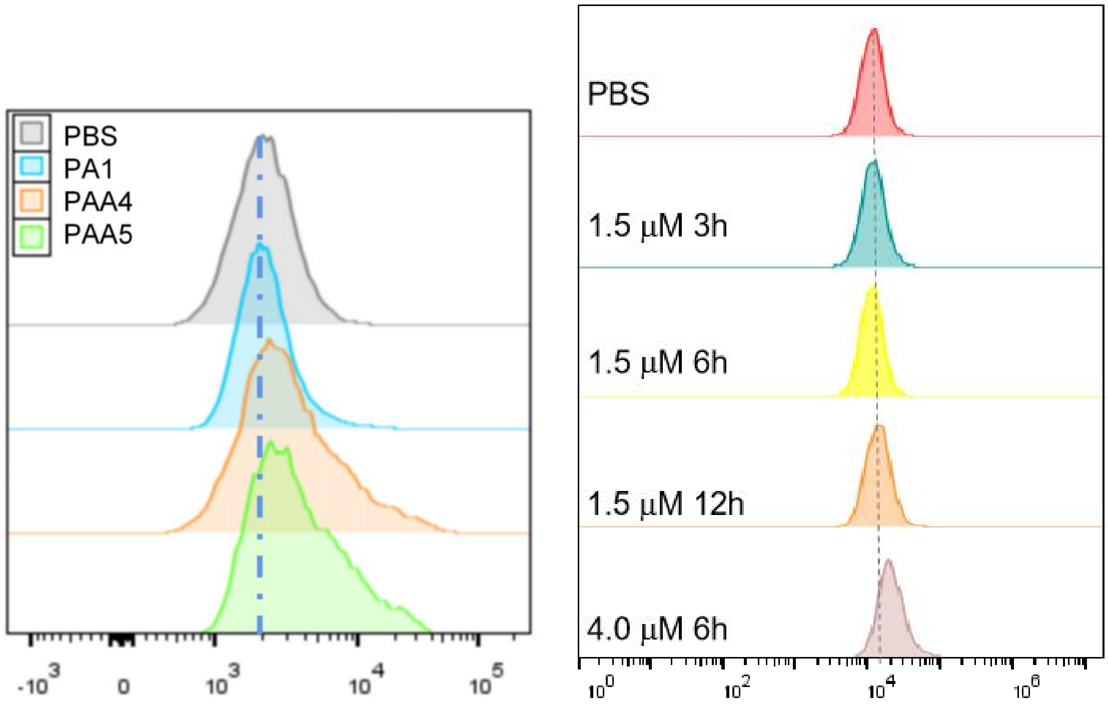

Supplementary Figure S25 Flow cytometry analysis of lipid peroxidation labeled by BODIPY-C11 in EJ cells treated by 8.0 μM **PA1**, 4.0 μM **PAA4** or 4.0 μM **PAA5** for 6h (Left). Lipid peroxidation increases in the **PAA4** and **PAA5**-treated EJ cells. Flow cytometry analysis of lipid peroxidation labeled by BODIPY-C11 in EJ cells treated by 1.5 μM 3h **PAA4**, 1.5 μM 6h **PAA4**, 1.5 μM 12h **PAA4** or 4.0 μM 6h **PAA4** (Right). (n = 3 independent experiments).

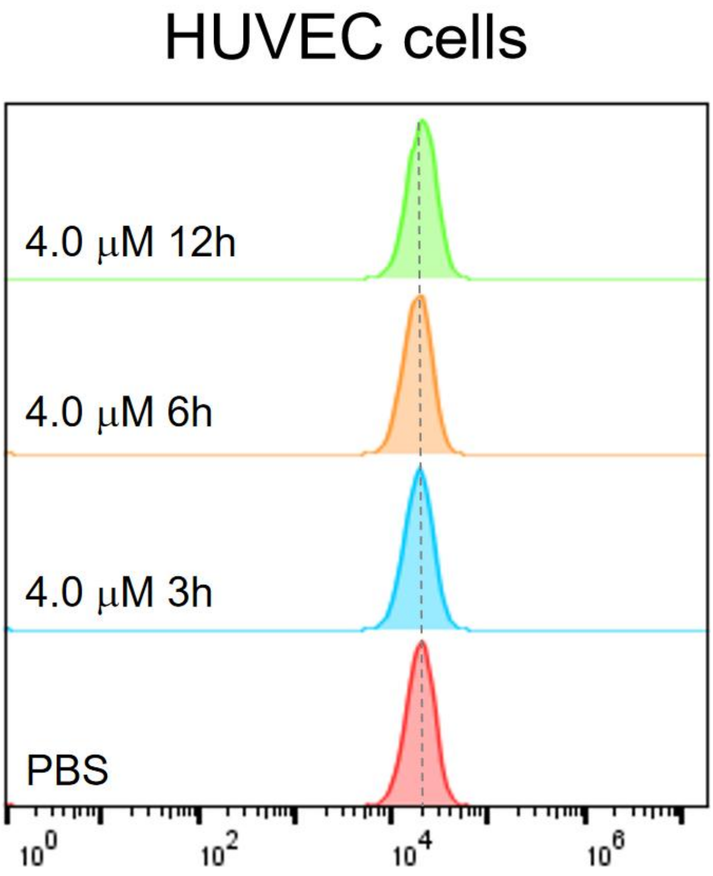

Supplementary Figure S26 Flow cytometry analysis of lipid peroxidation labeled by BODIPY-C11 in HUVEC cells treated by 4.0 μM **PAA4** for 3h, 6h and 12h. No lipid peroxidation was detected in the **PAA4**-treated HUVEC cells within 12h. (n = 3 independent experiments).

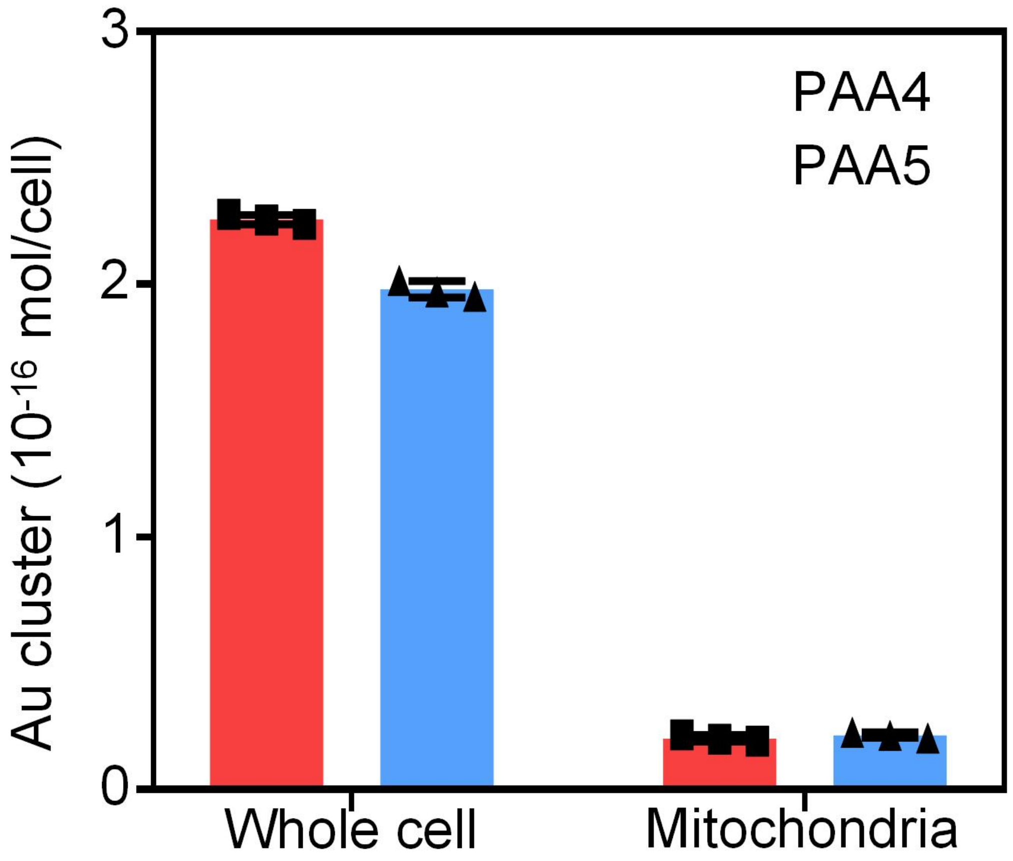

Supplementary Figure S27 Content of **PAA4** and **PAA5** in the whole EJ cells and cell-extracted mitochondria determined by ICP-MS ($c_{Au(I)\ clusters}$ = 1.5 μM, 24 h incubation). Data were expressed as mean ± SD (n = 3 independent experiments). Source data are provided as a Source Data file.

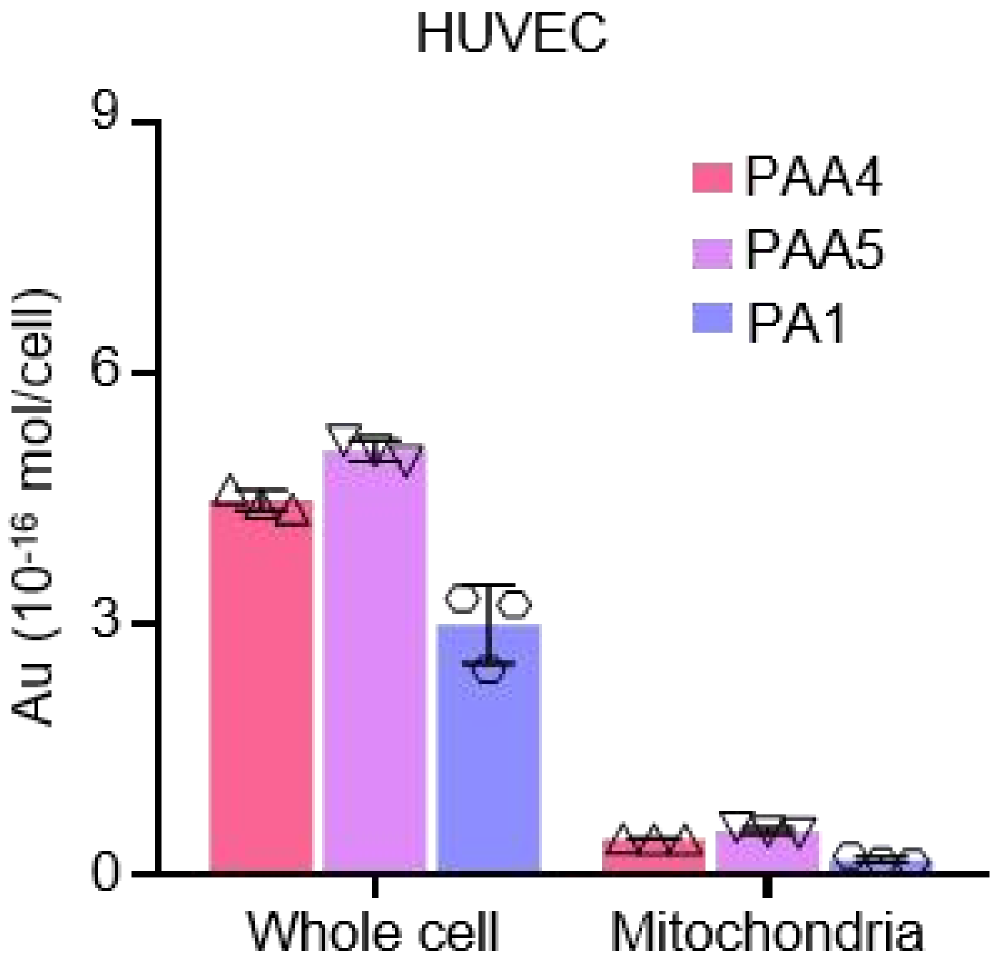

Supplementary Figure S28 Content of **PAA4**, **PAA5** and **PA1** in the whole HUVEC cells and cell-extracted mitochondria determined by ICP-MS ($c_{\mathrm{Au(I)\ clusters}}$ = 1.5 μM, $c_{\mathbf{PA1}}$ = 6.0 μM, 4 h incubation). Data were expressed as mean ± SD (n = 3 independent experiments). Source data are provided as a Source Data file.

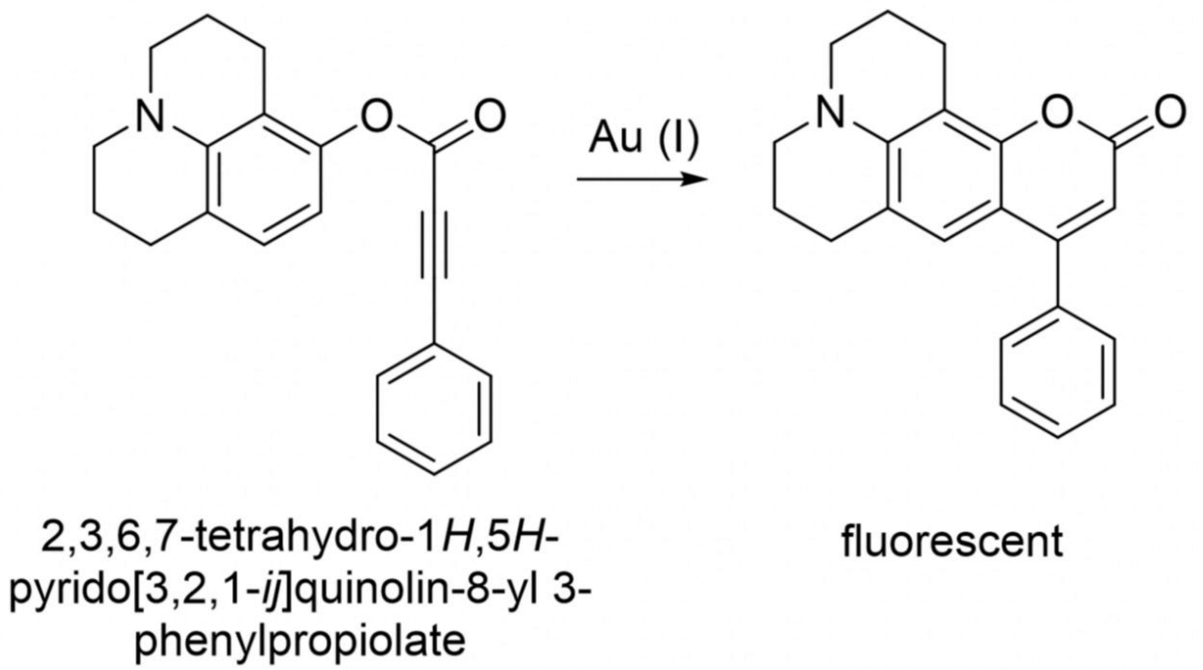

Supplementary Figure S29 Au(I)-catalyzed cyclization of 2,3,6,7-tetrahydro-1H,5H-pyrido[3,2,1-ij]quinolin-8-yl-3-phenylpropiolate to generate a fluorescent product excited at 488 nm.

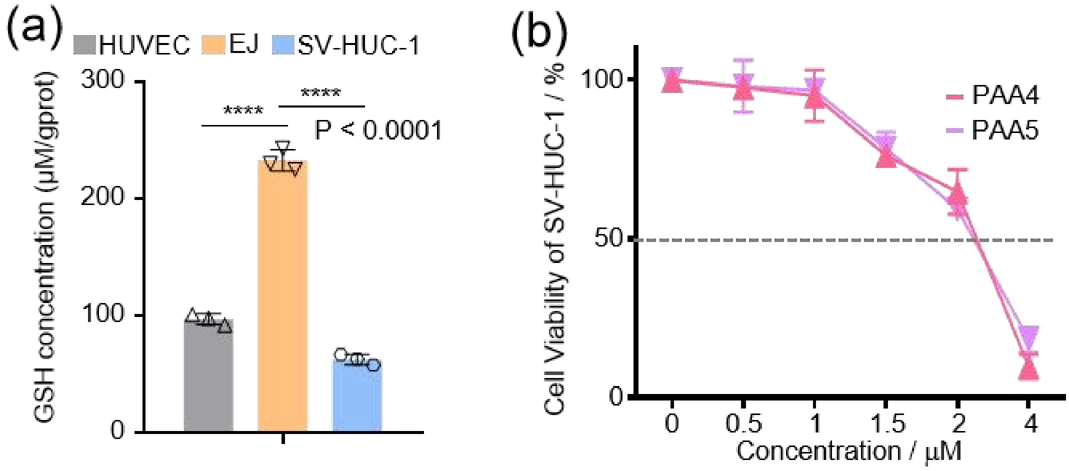

Supplementary Figure S30 (a) Intracellular GSH concentrations in HUVEC, EJ and SV-HUC-1 cells determined by GSH assay kits. (b) Cell viability assay of SV-HUC-1 cells treated with **PAA4** and **PAA5** for 24 h (IC50 2.3 μM for SV-HUC-1 cells). Asterisks (*) denote the statistical significance: 0.01< * $P < 0.05$, 0.001< ** $P \leq 0.01$, 0.0001< *** $P \leq 0.001$, **** $P \leq 0.0001$, P values were performed with one-way ANOVA followed by post hoc Tukey's test. Data were expressed as mean ± SD (n = 3 independent experiments examined over triplicates).Source data are provided as a Source Data file.

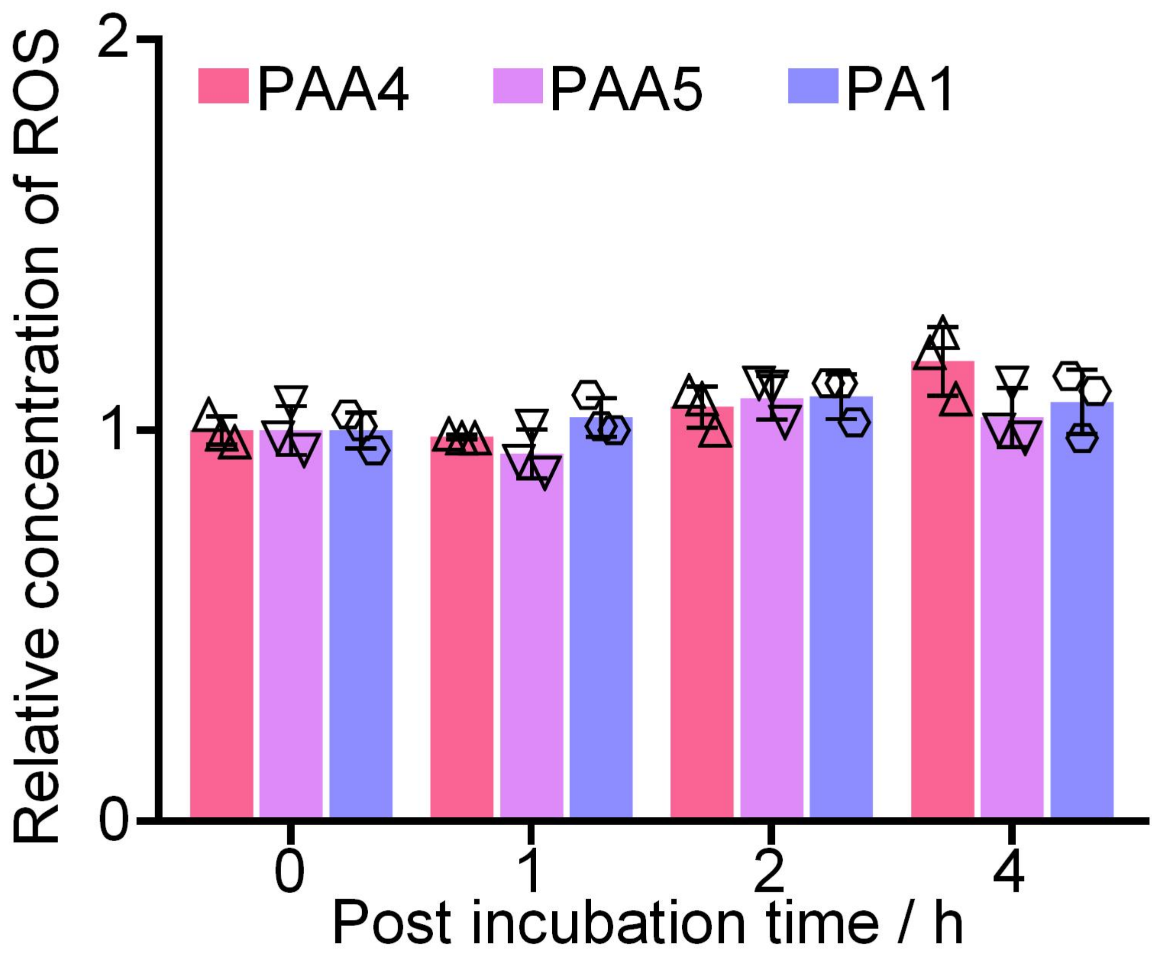

Supplementary Figure S31 Time-dependent intracellular ROS production changes in HUVEC cells treated by **PAA4**, **PAA5** (1.5 μM, 4 h incubation) and **PA1** (6.0 μM, 4 h incubation). Data were expressed as mean ± SD (n = 3 independent experiments examined over triplicates). ROS levels show no significant change in HUVEC cells treated by **PAA4**, **PAA5** or **PA1**. Source data are provided as a Source Data file.

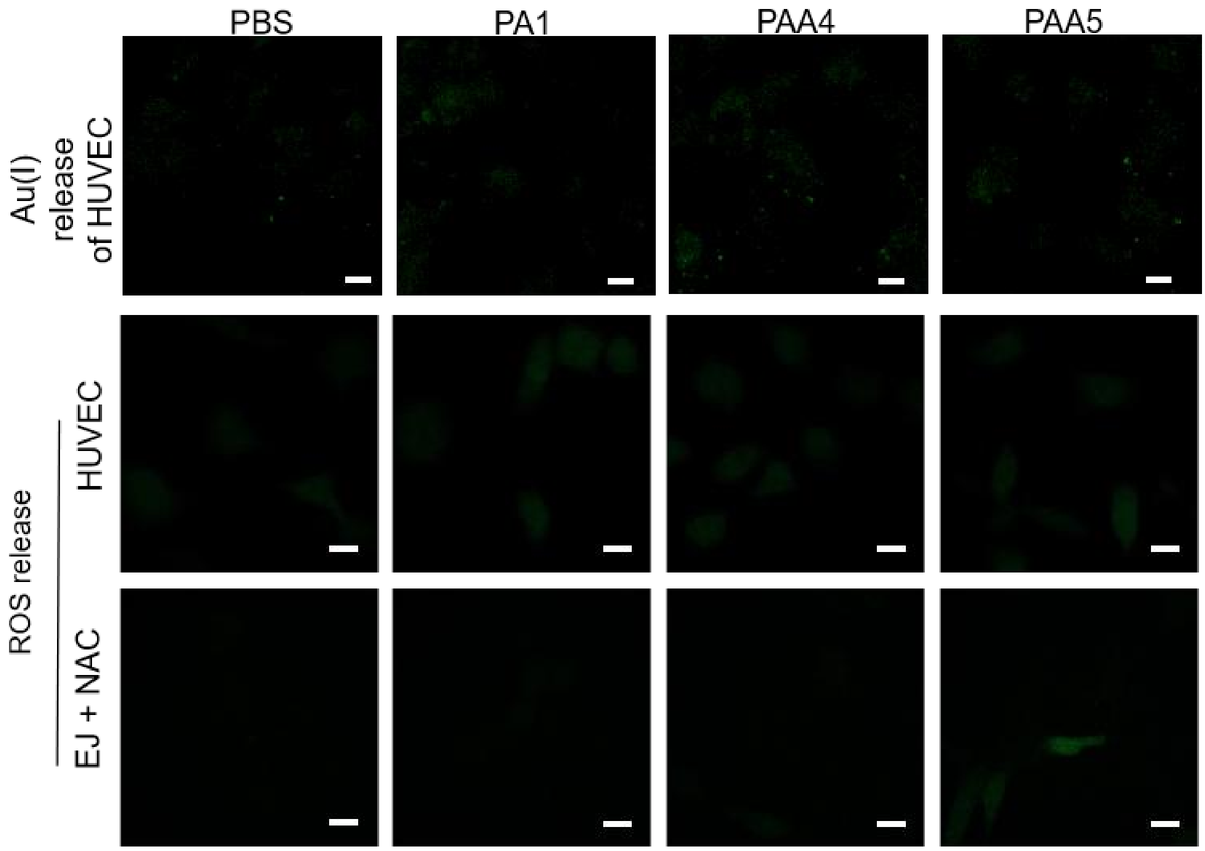

Supplementary Figure S32 Release of Au(I) ions monitored by confocal laser scanning microscopy in the 2,3,6,7-tetrahydro-1H,5H-pyrido[3,2,1-ij]quinolin-8-yl-3-phenylpropiolate stained HUVEC cells (up), and ROS release monitored by confocal laser scanning microscopy in the 2’,7’-dichlorofluorescin diacetate (DCFH-DA, 10.0 μM) stained HUVEC cells (middle) and NAC (3.0 mM) pretreated EJ cells (down) treated with **PAA4**, **PAA5** (1.5 μM, 4 h incubation) and **PA1** (6.0 μM, 4 h incubation) relative to the PBS blank trial. Scale bar: 20 mm. Excitation: 488 nm. n = 3 independent experiments.

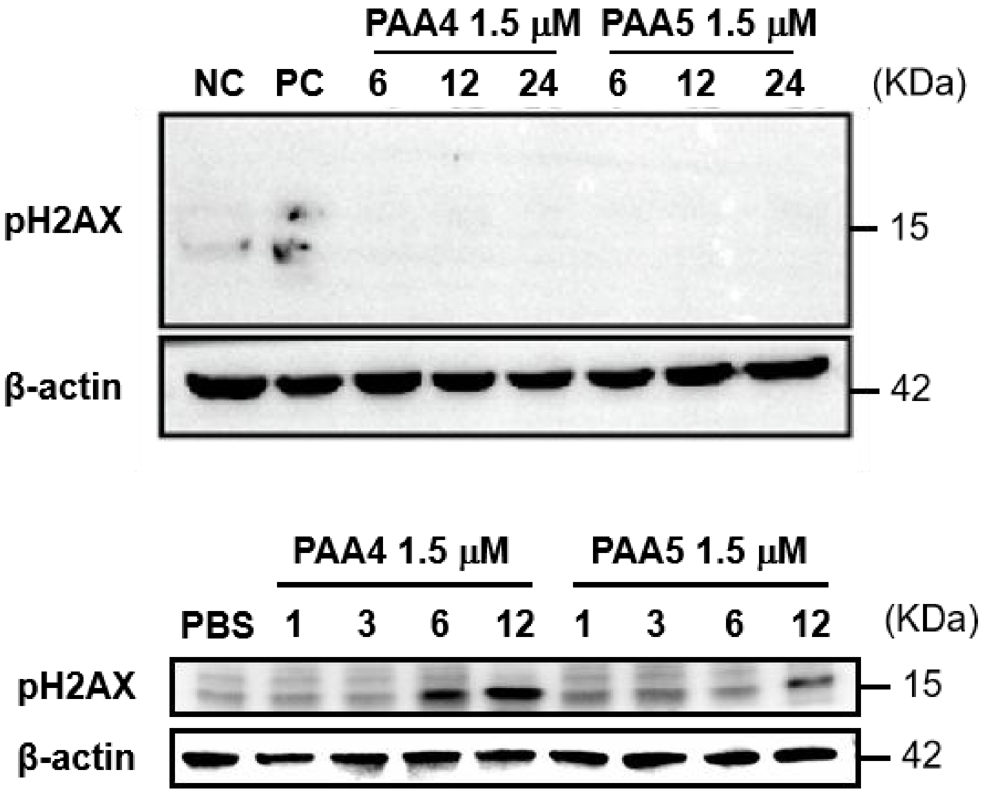

Supplementary Figure S33 Western blot analysis of pH2AX with 1.5 μ M dose of **PAA4/PAA5** in HUVECcells (up) for 24h and EJ cells (down) for 12h. n = 2 independent experiments. Source data are provided as a Source Data file.

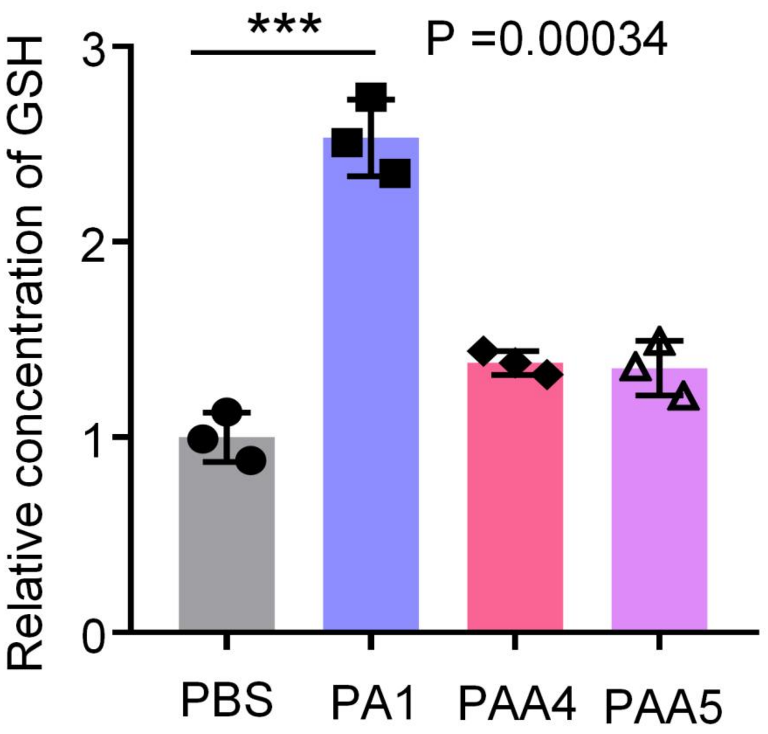

Supplementary Figure S34 Intracellular GSH concentrations in EJ cells upon the treatment with **PA1** (6.0 μM, 4 h incubation), **PAA4** (1.5 μM, 4 h incubation) and **PAA5** (1.5 μM, 4 h incubation) determined by GSH assay kits. Asterisks (*) denote the statistical significance: 0.01< * P < 0.05, 0.001< ** $P \leq 0.01$, 0.0001< *** $P \leq 0.001$, **** $P \leq 0.0001$, P values were performed with one-way ANOVA followed by post hoc Tukey's test. Data were expressed as mean ± SD (n = 3 independent experiments examined over triplicates). The GSH generation is only slightly increased in **PAA4** and **PAA5**-treated EJ cells due to a pro-oxidant response, comparing to siginificant over-expression of GSH in **PA1**-treated EJ cells. Source data are provided as a Source Data file.

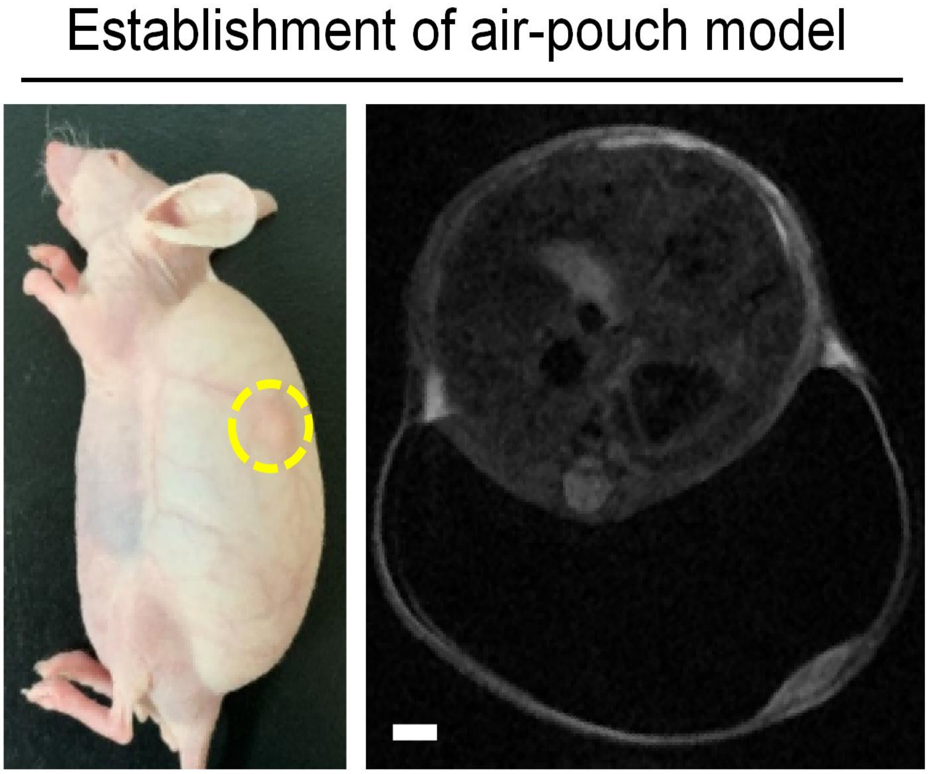

Supplementary Figure S35 Representative brighten field and Magnetic Resonance Imaging images of the tumor on the inner surface of the air-pouch. Scale bar: 0.5 cm. n=5 mice from a representative experiment.

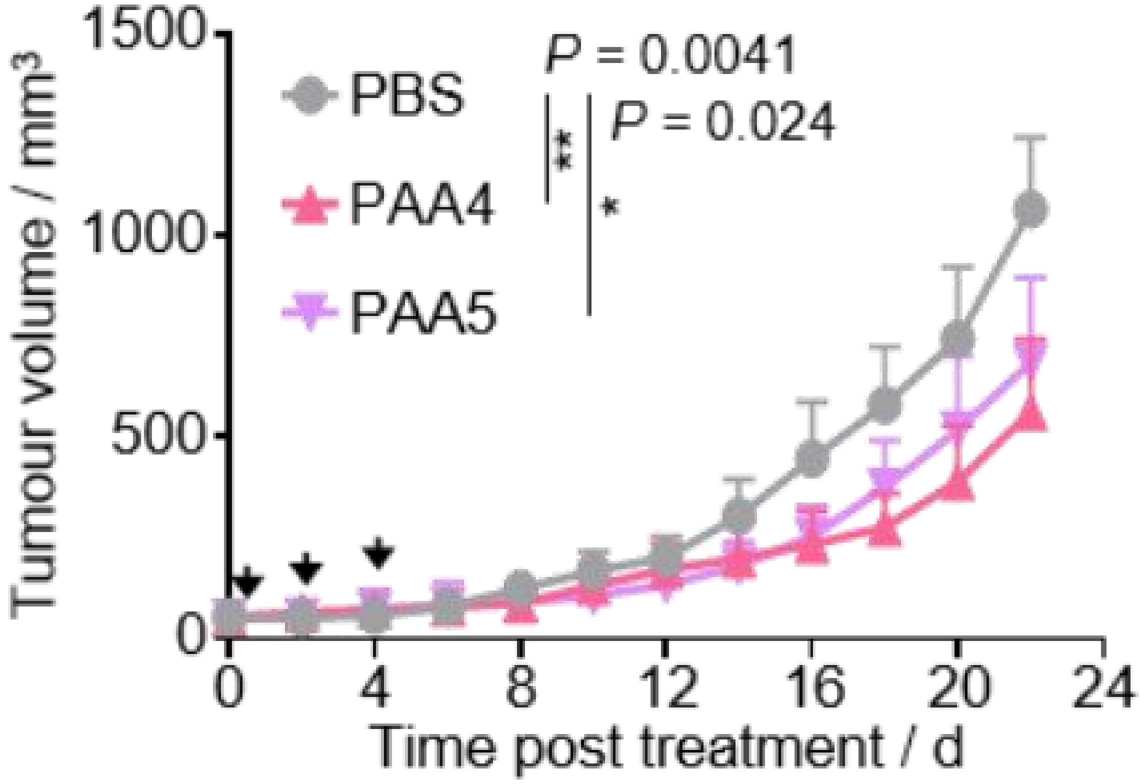

Supplementary Figure S36 Tumor growth curve (APBC model) after the treatment with PBS, **PAA4** (1.5 μM, 1 mL, 30 min) and **PAA5** (1.5 μM, 1 mL, 30 min). The black arrow indicates the administrated time point. The statistical comparison tumor growth curve was calculated on day 22. Asterisks (*) denote the statistical significance: 0.01 < * $P < 0.05$, 0.001< ** $P \leq 0.01$, 0.0001< *** $P \leq 0.001$, **** $P \leq 0.0001$, P values were performed with one-way ANOVA followed by post hoc Tukey's test. Data were expressed as mean ± SD (n=5 mice in 3 independent groups from a representative experiment). Source data are provided as a Source Data file.

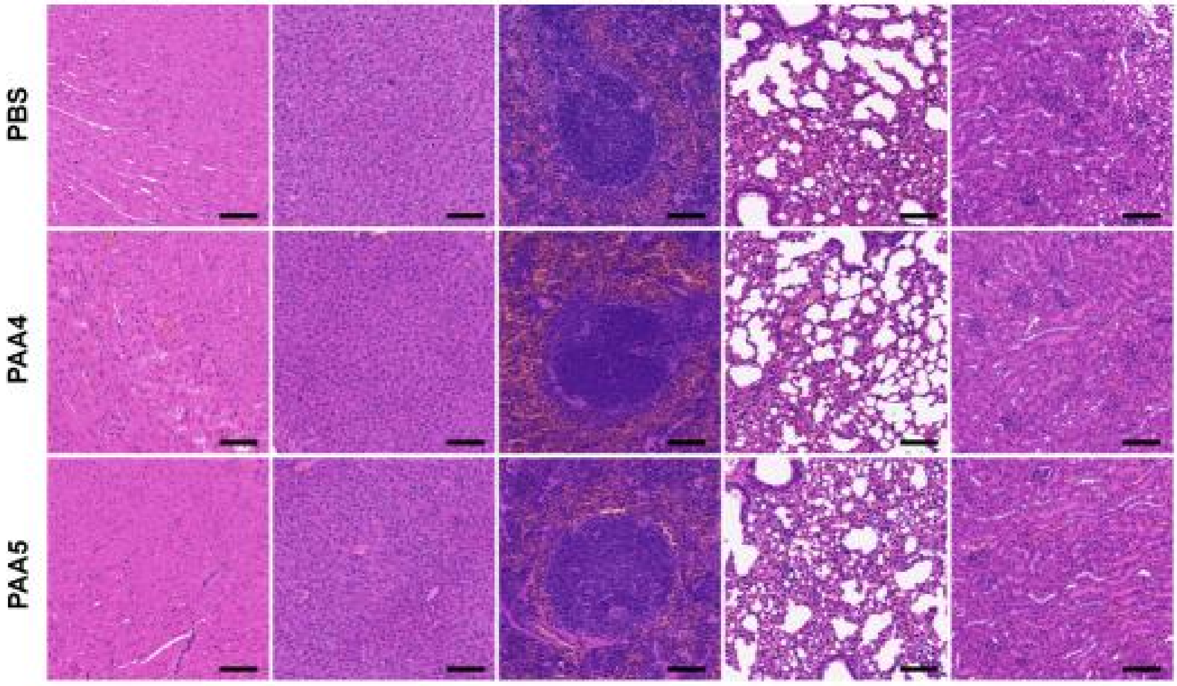

Supplementary Figure S37 Histology evaluation of the major organs (heart, liver, spleen, lung, and kidney, APBC model) collected from the PBS-, **PAA4-** and **PAA5**-treated group. Scale bar = 100 μm. No significant tissue toxicity is found. n=5 mice in 3 independent groups from a representative experiment.

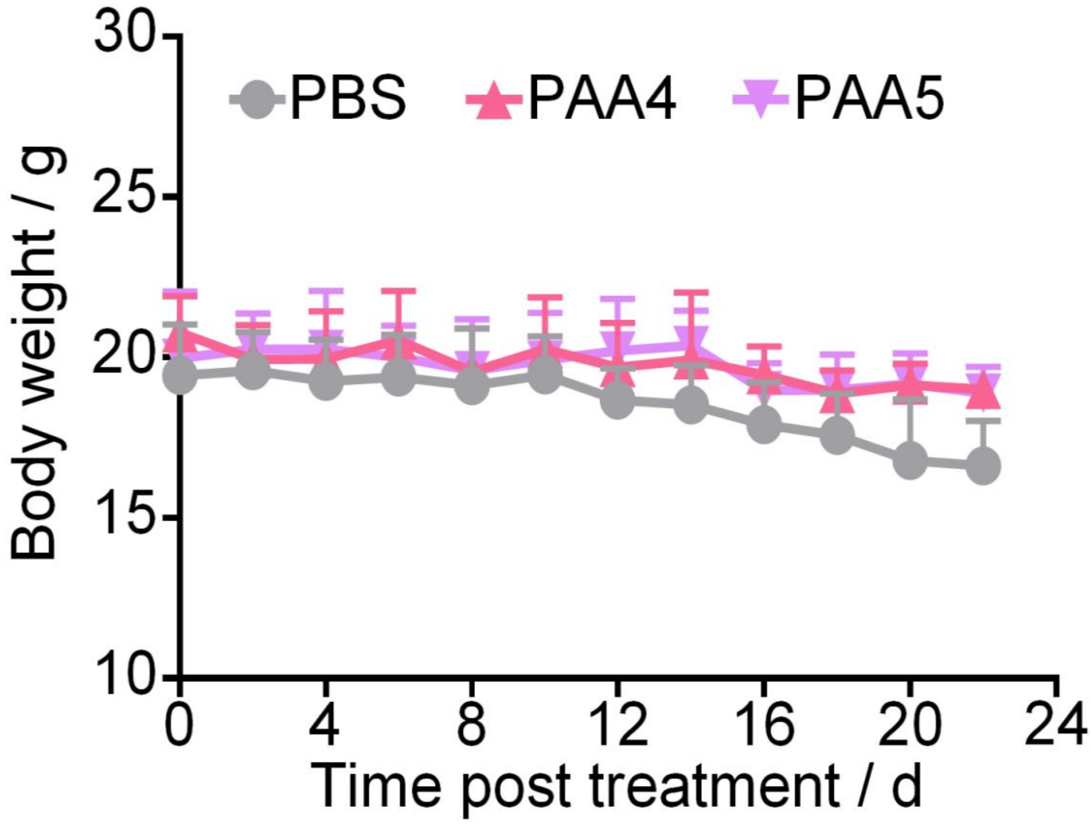

Supplementary Figure S38 Body weight changes of EJ APBC nude mice after the treatment with PBS, **PAA4** and **PAA5**. The body weight changes were monitored every two days for 22 days. Data are presented as the mean ± SD (n=5 mice in 3 independent groups from a representative experiment). Source data are provided as a Source Data file.

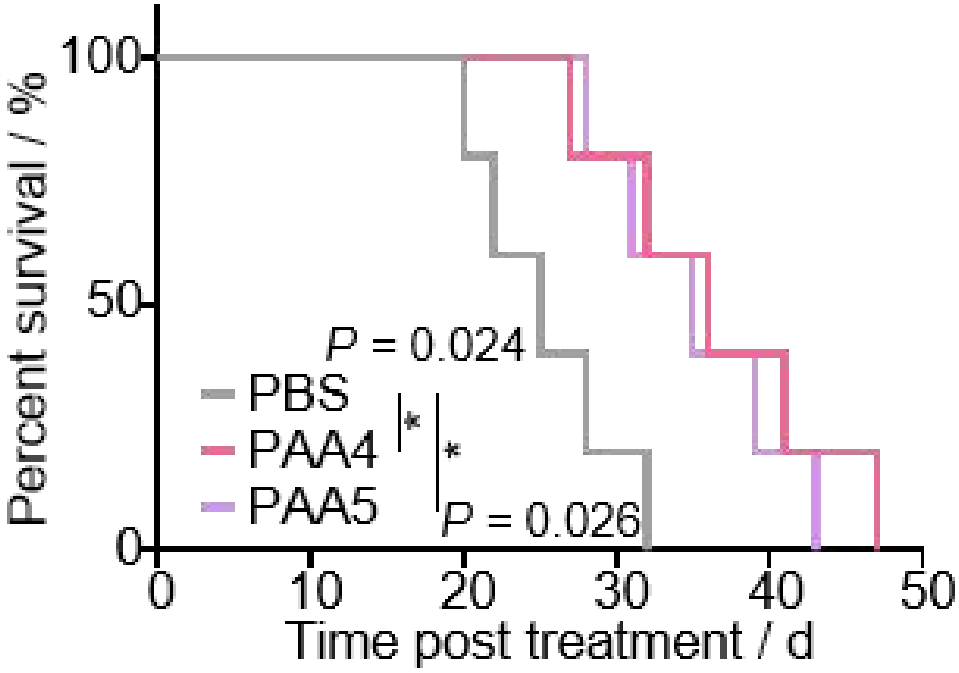

Supplementary Figure S39 Kaplan-Meimer survival curve of EJ APBC nude mice. Asterisks (*) denote the statistical significance: 0.01 < * P < 0.05, 0.001 < ** $P \leq 0.01$, 0.0001 < *** $P \leq 0.001$, **** $P \leq 0.0001$, P values were performed with one-way ANOVA followed by post hoc Tukey's test. Data were expressed as mean ± SD (n=5 mice in 3 independent groups from a representative experiment). Source data are provided as a Source Data file.

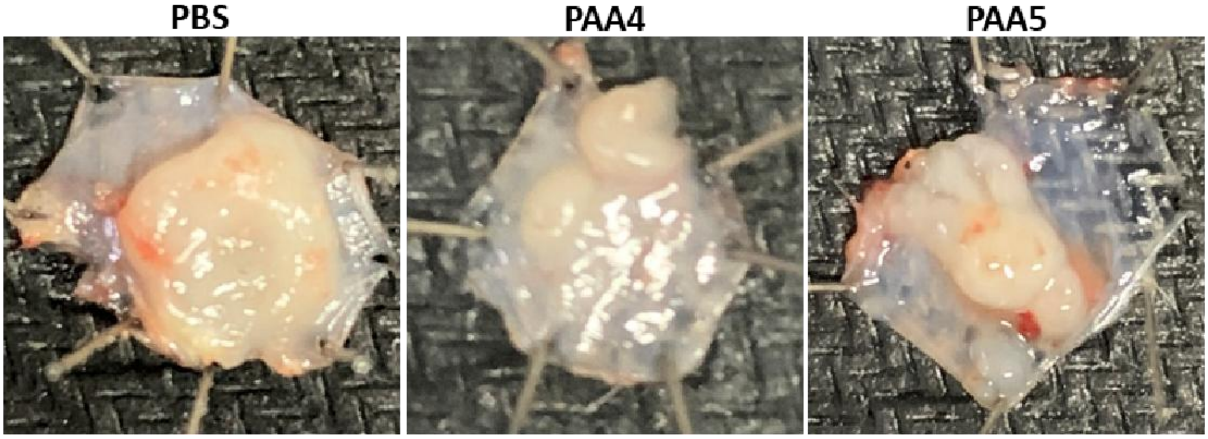

Supplementary Figure S40 Photography of mice bladders of PBS, **PAA4**-treated and **PAA5**-treated groups. n=5 mice from a representative experiment.

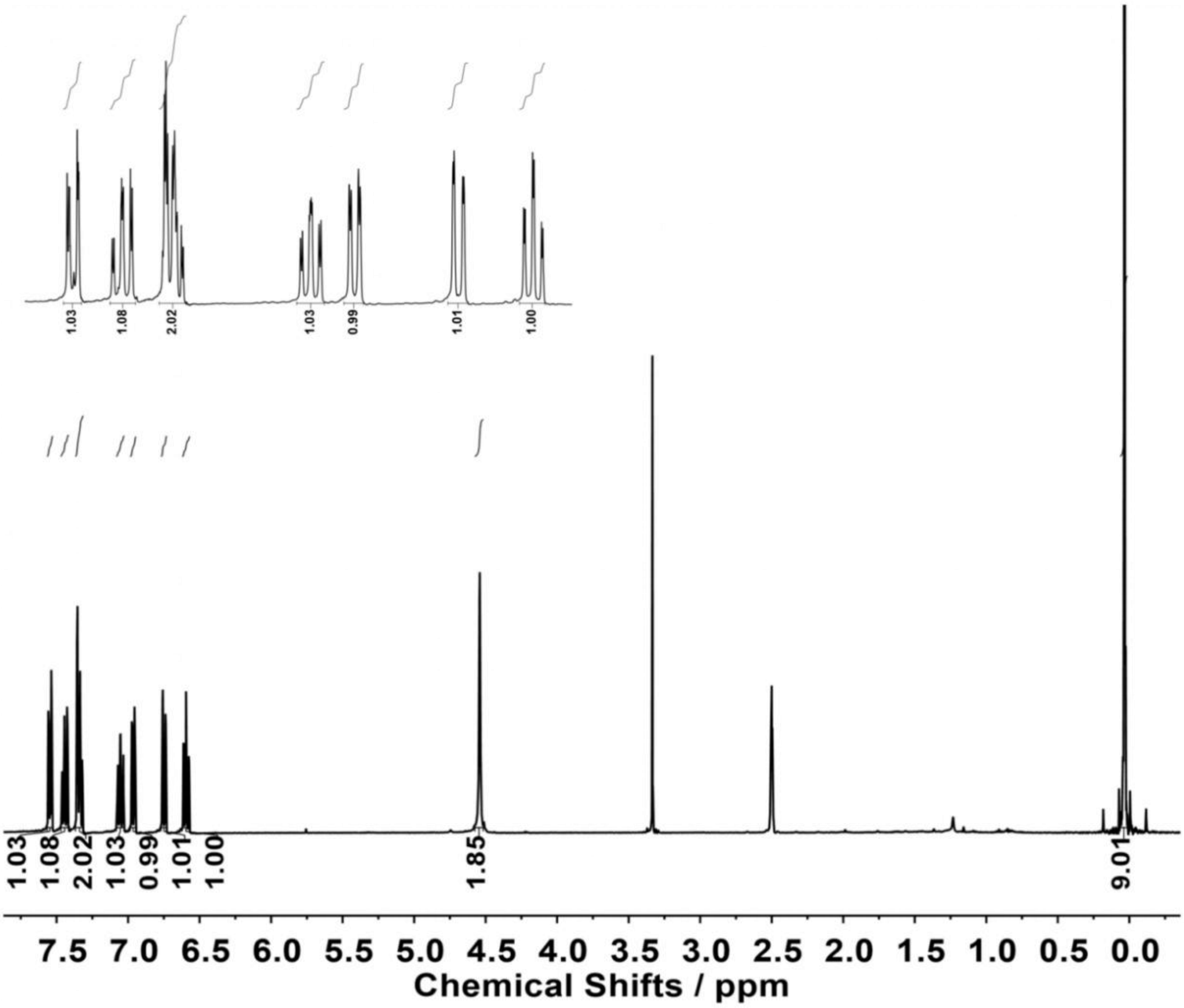

Supplementary Figure S41 ^{1}H NMR spectrum of **TEBA** (400 MHz, DMSO-d_6, 298K).

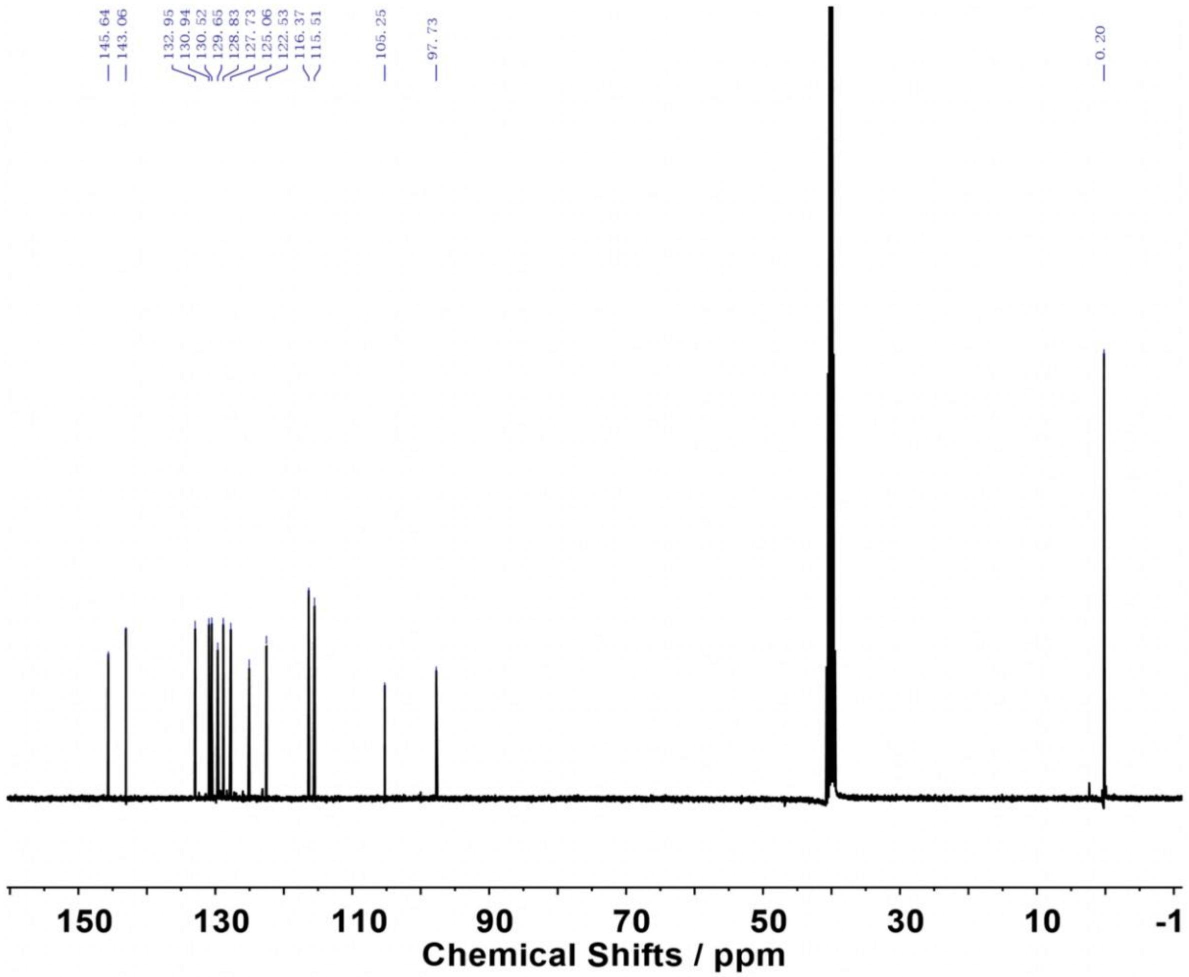

Supplementary Figure S42 ^{13}C NMR spectrum of **TEBA** (100 MHz, DMSO-d_6, 298K).

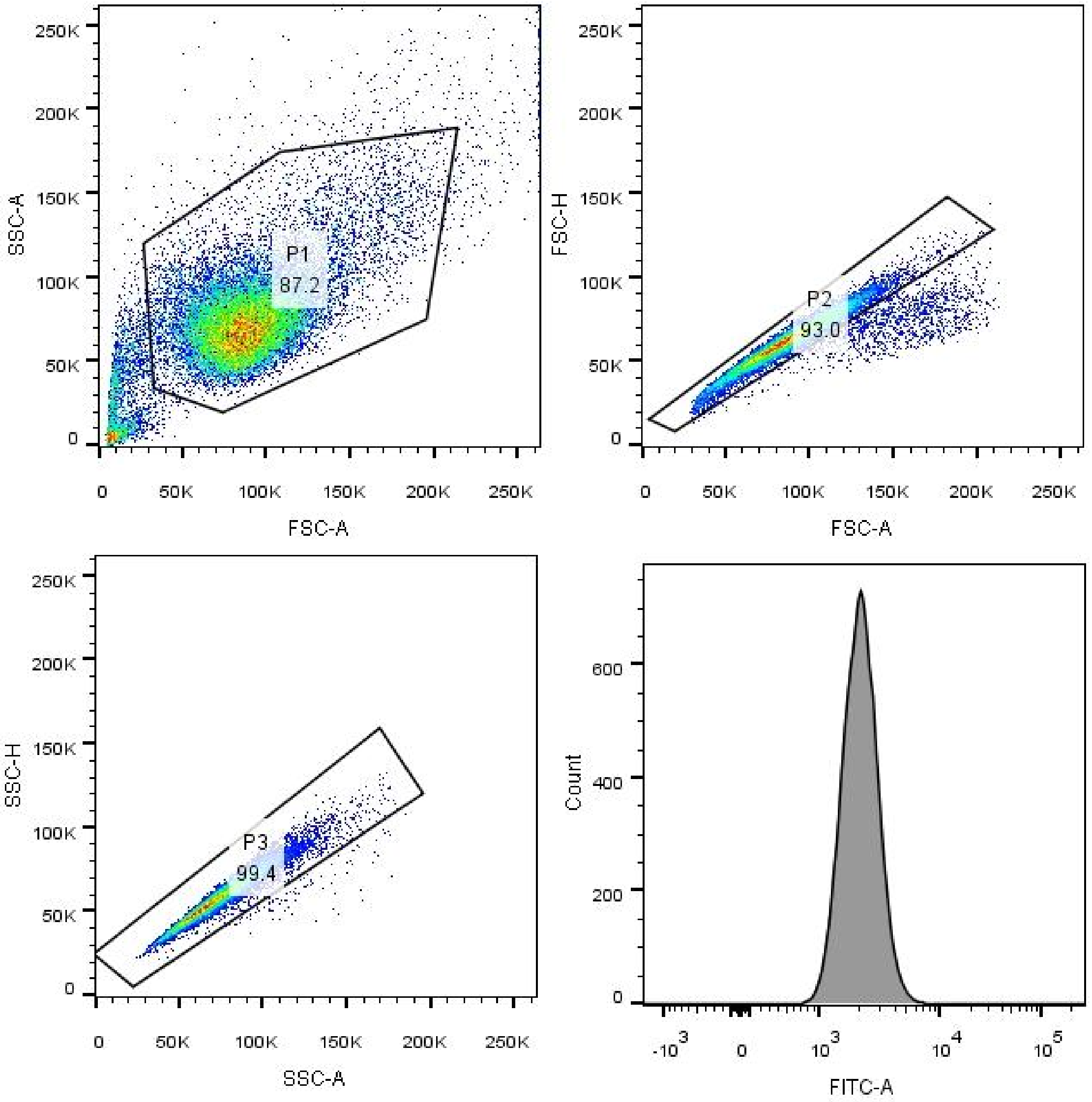

Supplementary Figure S43 Gating strategy for for EJ cells flow cytometry. All FACS files in the same experiment follow the same gating strategy.
