# Supplementary material for: Pro-oxidant response and accelerated ferroptosis caused by synergetic Au(I) release in hypercarbon-centered gold(I) cluster prodrugs
Source: Nat Commun. 2022 Aug 9;13:4669. doi: 10.1038/s41467-022-32474-y (PMC9363434; doi:10.1038/s41467-022-32474-y)
Supplement: Supplementary file 3 — Reporting Summary [file 41467_2022_32474_MOESM3_ESM.pdf]

nature portfolio

Corresponding author(s): Liang Zhao
Manuscript number:NCOMMS-21-13435B

Last updated by author(s): Jul 12, 2022

Reporting Summary

Nature Portfolio wishes to improve the reproducibility of the work that we publish. This form provides structure for consistency and transparency in reporting. For further information on Nature Portfolio policies, see our Editorial Policies and the Editorial Policy Checklist.

Statistics

For all statistical analyses, confirm that the following items are present in the figure legend, table legend, main text, or Methods section.

n/a	Confirmed	
☐	☒	The exact sample size (n) for each experimental group/condition, given as a discrete number and unit of measurement
☐	☒	A statement on whether measurements were taken from distinct samples or whether the same sample was measured repeatedly
☐	☒	The statistical test(s) used AND whether they are one- or two-sided<br>*Only common tests should be described solely by name; describe more complex techniques in the Methods section.*
☒	☐	A description of all covariates tested
☒	☐	A description of any assumptions or corrections, such as tests of normality and adjustment for multiple comparisons
☐	☒	A full description of the statistical parameters including central tendency (e.g. means) or other basic estimates (e.g. regression coefficient) AND variation (e.g. standard deviation) or associated estimates of uncertainty (e.g. confidence intervals)
☐	☒	For null hypothesis testing, the test statistic (e.g. F, t, r) with confidence intervals, effect sizes, degrees of freedom and P value noted<br>*Give P values as exact values whenever suitable.*
☒	☐	For Bayesian analysis, information on the choice of priors and Markov chain Monte Carlo settings
☒	☐	For hierarchical and complex designs, identification of the appropriate level for tests and full reporting of outcomes
☒	☐	Estimates of effect sizes (e.g. Cohen's d, Pearson's r), indicating how they were calculated

Our web collection on statistics for biologists contains articles on many of the points above.

Software and code

Policy information about availability of computer code

Data collection	Confocal laser scanning microscopy (UltraVIEW VoX), Zeiss 710; in vivo imaging system (IVIS Spectrum); Immunofluorescence (Perkin Elmer Operetta HCS); Blood biochemistry and haematology analyses (Vital River Laboratory Animal Technology Co. Ltd).1H, 13C and 31P NMR were carried out on a JEOL ECX-400 MHz instrument. The UV light irradiation experiment was carried out using Agilent Cary Series UV-vis-NIR. High resolution mass spectra were obtained on a Thermo Scientific Exactive Orbitrap instrument with an ESI mode.
Data analysis	Bar graph, curves and the relevant statistics were analyzed by GraphPad Prism 8.0 and origin 9.0; Confocal data were analyzed by Volocity Demo; Western blot images were analyzed by ImageJ 1.52g. X-ray crystallographic analysis were processed by SHELXTL program and Olex2 (v1.3) program. All crystal imaging were processed by using X-seed (v1.5) program.

For manuscripts utilizing custom algorithms or software that are central to the research but not yet described in published literature, software must be made available to editors and reviewers. We strongly encourage code deposition in a community repository (e.g. GitHub). See the Nature Portfolio guidelines for submitting code & software for further information.

Data

Policy information about availability of data

All manuscripts must include a data availability statement. This statement should provide the following information, where applicable:

- Accession codes, unique identifiers, or web links for publicly available datasets
- A description of any restrictions on data availability
- For clinical datasets or third party data, please ensure that the statement adheres to our policy

The X-ray crystallographic coordinates for structures reported in this article have been deposited at the Cambridge Crystallographic Data Centre (CCDC), under deposition number CCDC-2003898 (PAA4) and 2003897 (PAA5). These data can be obtained free of charge from the Cambridge Crystallographic Data Centre via

www.ccdc.cam.ac.uk/data_request/cif. For full characterization data including NMR and ESI-MS spectra and experimental details, see the Supplemental Information. Source data are provided with this paper. The remaining data are available within the Article, Supplementary Information or Source Data file.

Field-specific reporting

Please select the one below that is the best fit for your research. If you are not sure, read the appropriate sections before making your selection.

[x] Life sciences   [ ] Behavioural & social sciences   [ ] Ecological, evolutionary & environmental sciences

For a reference copy of the document with all sections, see nature.com/documents/nr-reporting-summary-flat.pdf

Life sciences study design

All studies must disclose on these points even when the disclosure is negative.

Sample size	We choose six mice in each group for treatment, which is representative of treatment outcomes. Sample sizes were based on our previous experience and other publications, providing enough statistical power to detect the usually strong effects observed in our experiments. All sample sizes are clearly described in the manuscript or the figure legends.
Data exclusions	No data were excluded from the analyses.
Replication	All the experiments were performed in triplicates with a good reproducibility of the experimental findings.
Randomization	All samples were randomly allocated into different groups.
Blinding	The investigators were blinded to group allocation during data collection and/or analysis.

Reporting for specific materials, systems and methods

We require information from authors about some types of materials, experimental systems and methods used in many studies. Here, indicate whether each material, system or method listed is relevant to your study. If you are not sure if a list item applies to your research, read the appropriate section before selecting a response.

Materials & experimental systems

n/a	Involved in the study	
[ ]	[x]	Antibodies
[ ]	[x]	Eukaryotic cell lines
[x]	[ ]	Palaeontology and archaeology
[ ]	[x]	Animals and other organisms
[x]	[ ]	Human research participants
[x]	[ ]	Clinical data
[x]	[ ]	Dual use research of concern

Methods

n/a	Involved in the study	
[x]	[ ]	ChIP-seq
[ ]	[x]	Flow cytometry
[x]	[ ]	MRI-based neuroimaging

Antibodies

Antibodies used	beta actin antibody：from Abcepta,　cat：AM1021B,　Lot：SG190114AA；<br>pH2AX antibody： from millipore, Cat：05-636. Lot：1997719；<br>PARP antibody： from CST, cat：#9542， Lot：9；<br>Anti-Caspase-3 antibody: from abcam, cat: ab184787;<br>Anti-Bax antibody: from abcam, cat: ab32503;<br>Anti-Bcl-2 antibody: from abcam, cat: ab182858;<br>PTGS2 antibody：from Abclonal,　cat：A3560,　Lot：4000000800.
Validation	beta actin antibody suitable for: WB, IHC-P, E; reactivity: Human, Mouse, rat;<br>pH2AX antibody suitable for: WB, IHC, etc; reactivity: vertebrates;<br>PARP antibody suitable for: WB; reactivity: Human Mouse Rat Mk<br>PTGS2 antibody suitable for: WB,　IHC,　reactivity：Human, Mouse, Rat;<br>Information from manufacture's website.<br>Anti-Caspase-3 antibody suitable for: WB, IHC-P, IP;<br>Anti-Bax antibody suitable for: IHC-P, WB, IP, Sandwich ELISA;<br>Anti-Bcl-2 antibody suitable for: Flow Cyt (Intra), WB, IHC-P.

Eukaryotic cell lines

Policy information about cell lines

Cell line source(s)	EJ, HUVEC and SV-HUC-1 cell lines were purchased from National Infrastructure of Cell Line Resource.
Authentication	None of the cell lines used were authenticated.
Mycoplasma contamination	The cell lines were not tested for mycoplasma contamination.
Commonly misidentified lines (See ICLAC register)	No commonly misidentified cell lines were used in the study.

Animals and other organisms

Policy information about studies involving animals; ARRIVE guidelines recommended for reporting animal research

Laboratory animals	Female BALB/c nude mice (4-6 weeks, about 18 g); Female BALB/c nude mice (6–8 weeks, 16–18 g) were purchased from Vital River Laboratory Animal Technology Co., Ltd. (Beijing, China).
Wild animals	No wild animals were used in the study.
Field-collected samples	No field collected samples were used in the study.
Ethics oversight	All animal experiments were performed in accordance with the Guide for Care and Use of Laboratory Animals, approved by the Committee for Animal Research of National Center for Nanoscience and Technology, China. Issue No. NCNST21-2011-0602.

Note that full information on the approval of the study protocol must also be provided in the manuscript.

Flow Cytometry

Plots

Confirm that:

- [x] The axis labels state the marker and fluorochrome used (e.g. CD4-FITC).
- [x] The axis scales are clearly visible. Include numbers along axes only for bottom left plot of group (a 'group' is an analysis of identical markers).
- [x] All plots are contour plots with outliers or pseudocolor plots.
- [x] A numerical value for number of cells or percentage (with statistics) is provided.

Methodology

Sample preparation	EJ cells were treated with different compounds accordingly, labeled with dye, washed with PBS, digested with trypsin, spinned down, and resuspend with PBS for flow cytometry analysis.
Instrument	Cells were analyzed on BD LSRFortessa SORP（BD）
Software	Data were collected on BD FACSDiva™ Software, and analyzed using Flowjo V10
Cell population abundance	No sorting were used in this study.
Gating strategy	Single cell population was determined by FSC-A/FSC-H gate, followed by a SSC-A/SSC-H gate. And the boundaries were determined by the clear cell subgroups.

- [x] Tick this box to confirm that a figure exemplifying the gating strategy is provided in the Supplementary Information.
